# Linker histone H1.2 establishes chromatin compaction and gene silencing through recognition of H3K27me3

**DOI:** 10.1038/srep16714

**Published:** 2015-11-19

**Authors:** Jin-Man Kim, Kyunghwan Kim, Vasu Punj, Gangning Liang, Tobias S. Ulmer, Wange Lu, Woojin An

**Affiliations:** 1 Department of Biochemistry and Molecular Biology, University of Southern California, Los Angeles, CA 90033, USA; 2 Norris Comprehensive Cancer Center, University of Southern California, Los Angeles, CA 90033, USA; 3 Department of Medicine, University of Southern California, Los Angeles, CA 90033, USA; 4 Department of Urology, Keck School of Medicine, University of Southern California, Los Angeles, CA 90033, USA; 5 Zilkha Neurogenetic Institute, Keck School of Medicine, University of Southern California, Los Angeles, CA 90033, USA; 6 Eli and Edythe Broad Center for Regenerative Medicine and Stem Cell Research, University of Southern California, Los Angeles, CA 90033, USA; 7 Department of Biology, College of Natural Sciences, Chungbuk National University, Cheongju, Chungbuk 361-763, Republic of Korea

## Abstract

Linker histone H1 is a protein component of chromatin and has been linked to higher-order chromatin compaction and global gene silencing. However, a growing body of evidence suggests that H1 plays a gene-specific role, regulating a relatively small number of genes. Here we show that H1.2, one of the H1 subtypes, is overexpressed in cancer cells and contributes to gene silencing. H1.2 gets recruited to distinct chromatin regions in a manner dependent on EZH2-mediated H3K27me3, and inhibits transcription of multiple growth suppressive genes via modulation of chromatin architecture. The C-terminal tail of H1.2 is critical for the observed effects, because mutations of three H1.2-specific amino acids in this domain abrogate the ability of H1.2 to bind H3K27me3 nucleosomes and inactivate target genes. Collectively, these results provide a molecular explanation for H1.2 functions in the regulation of chromatin folding and indicate that H3K27me3 is a key mechanism governing the recruitment and activity of H1.2 at target loci.

Genomic DNA in eukaryotic cells is stored in the nucleus via its hierarchical compaction into chromatin. The basic unit of chromatin is the nucleosome, in which approximately 147 base pairs of DNA are wrapped around a core histone octamer composed of H2A, H2B, H3, and H4^1^,^2^. Linker histone H1 is an additional histone protein that binds to the nucleosome at the site where internucleosomal DNA enters and exits the nucleosome core particle^3^. H1 plays an important structural role by folding nucleosomes into a higher-order chromatin structure known as the 30 nm chromatin fiber^4^. Metazoan histone H1 has a tri-partite structure that consists of a short unstructured N-terminal tail, a highly conserved central globular domain, and a long positively charged C-terminal tail^5^. Another notable characteristic of linker histone H1 is its high heterogeneity, as most species contain multiple H1 variants^3^,^5^. Most of these H1 subtypes are very highly conserved in the central globular core domain but display some degree of sequence heterogeneity in the N- and C-terminal tails^6^.

Early *in vitro* studies indicated that H1 binding to chromatin impairs transcription events by stabilizing the nucleosome, controlling nucleosome spacing, and/or folding nucleosome arrays into 30 nm chromatin fiber^7^,^8^. However, more recent studies characterizing H1 subtypes challenge this original view and suggest that H1 is not a global repressor of transcription but rather plays a more dynamic and gene-targeted role, contributing to gene-specific transcriptional regulation^9^,^10^,^11^. For example, gene expression profiling to analyze the effects of knockout or knockdown of H1 subtypes reported that individual subtypes are involved in both up- and down-regulation of relatively small number of genes, rather than generating changes in global gene expression^9^,^10^,^11^. Although how H1 subtypes play such a specific regulatory role in gene transcription is unclear, amino acid sequence divergence in the tail regions of H1 subtypes seems to increase their functional specialization. Additionally, genome-wide mapping revealed that H1 subtypes are not uniformly distributed along the genome and that they are enriched at different genomic regions^11^,^12^,^13^,^14^. This provides a possible connection between the abundance of H1 subtypes and the silencing of specific genes.

Related to the current report, EZH2 is the catalytic subunit of the Polycomb repressive complex 2 (PRC2) that mediates H3K27 trimethylation (H3K27me3) and controls transcriptional activity at target loci^15^. In the prevailing model, the local enrichment of EZH2-mediated H3K27me3 promotes the recruitment of another polycomb complex, PRC1, thus stimulating monoubiquitylation of H2AK119 (H2AK119ub) and repressing gene transcription via the inhibition of RNAPII activation^16^,^17^. This hierarchical model would predict that PRC1 and PRC2 largely occupy a common set of genes, as indeed shown by several genome-wide studies^18^,^19^. However, in contrast to this original view, PRC1 recruitment and H2AK119ub at target genes could be achieved in the absence of EZH2 and in H3K27me3-independent manner^20^,^21^. More recent studies also discovered that PRC1-dependent H2AK119ub plays a critical role in PRC2 occupancy and H3K27me3 at target sites^22^. These results leave open the interesting question of whether any other factors act as a key regulator of PRC2-mediated gene silencing and whether H3K27me3 is coordinated with the action of other chromatin factors. Importantly, EZH2 is overexpressed or mutated in several human cancers, and is linked to the initiation of tumorigenesis through a variety of mechanisms, which ultimately prevent the expression of tumor suppressor genes^23^,^24^. Therefore, identification of novel downstream effectors of H3K27me3 signaling pathway will help uncover the molecular basis of gene silencing in cancer cells.

Here we identify and characterize H1.2, one of the H1 subtypes, as a new effector protein that recognizes EZH2-mediated H3K27me3 to trigger chromatin compaction and gene silencing in cancer cells. We show that the flexible C-terminal tail is essential for the high affinity binding of H1.2 to H3K27me3-enriched chromatin and the establishment of inactive transcription state. Supporting these results, our transcriptome analyses of cancer cells demonstrate that multiple growth suppressive genes are re-activated upon knockdown of H1.2 and EZH2, and that more than half of the genes up-regulated after H1.2 knockdown are also trans-activated in response to EZH2 knockdown.

## Results

### H1.2 selectively interacts with H3K27me3 nucleosomes

**Figure 1:**
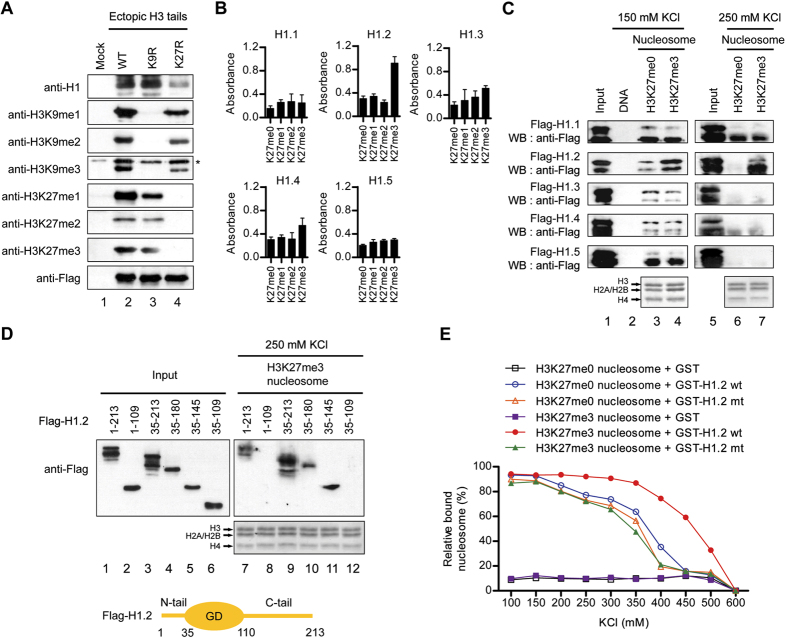
H1.2 binding to H3K27me3 nucleosomes *in vitro*. (**A**) Wild type (WT) and mutant (K9R and K27R) versions of Flag-tagged H3 tails were expressed in 293T cells and subjected to immunoprecipitation using anti-Flag antibody. The purified samples were resolved on 10% SDS-PAGE, and the presence of H1 and the methylation of ectopic H3 tails at K9 and K27 were determined by Western blot. The asterisk indicates a non-specific band. (**B**) The biotinylated H3 tail peptides that were either unmethylated or mono-, di- or tri-methylated at K27 were immobilized onto streptavidin-coated 96-well plates and incubated with Flag-tagged H1 subtypes. After extensive washing, the binding of H1 subtypes to the H3 tail peptides was determined quantitatively by using a microplate reader. Data represent the means ± SD of three independent experiments. (**C**) Nucleosomes were reconstituted on a 207 bp 601 nucleosome positioning sequence using H3K27me0 or H3K27me3 histone octamers and immobilized on streptavidin beads. Flag-H1 subtypes were incubated with immobilized nucleosomes under150 mM and 250 mM KCl conditions, and their binding to nucleosomes was analyzed by Western blot. Lane 1 represents 10% of the input. (**D**) After incubation with H3K27me3 nucleosomes, the binding of H1.2 deletion mutants to nucleosomes was determined by Western blot. Input corresponds to 10% of H1.2 proteins used in the binding reactions. (**E**) H3K27me0 and H3K27me3 nucleosomes were reconstituted on FITC-labeled 601 positioning sequence and incubated with GST-H1.2 wild type (wt) or GST-H1.2 V120/T126/V132 mutant (mt). GST-H1.2 proteins were immobilized on glutathione-Sepharose beads under the indicated KCl concentrations, and their interaction with nucleosomes were assessed by fluorescence measurements of both supernatant and pellet. Data shown are representative of three independent experiments.

In our earlier study, we purified proteins capable of binding to histone H3 tails by ectopically expressing the first 40 amino acids of human histone H3^25^. This approach allowed us to identify multiple regulatory factors that can specifically associate with H3 tails *in vivo*. In these experiments, we noticed that ectopic H3 tails undergo dynamic cellular modifications and that linker histone H1 interacts with the modified H3 tails. To determine whether any particular tail modifications are required for the observed interaction with H1, we transfected 293T cells with an empty vector or expression vectors for wild type H3 tails or H3 tails individually mutated at K4, K9, K14, K18, K23 and K27. After confirming that wild type and mutant H3 tails were expressed at similar levels (data not shown), ectopic H3 tails and their bound proteins were purified from nuclear extracts by immunoprecipitation with anti-Flag antibody. Western blotting of wild type H3 tail-associated proteins with H1 antibody detected two bands: a faster migrating band corresponding to H1.0 and a slower migrating band corresponding to H1.1-H1.5 which are replication-dependent somatic linker histones (Fig. 1A). When K4, K9, K14, K18, K23 were mutated to block their acetylation (at K9, K14, K18 and K23) or methylation (at K4 and K9), there were no detectable effects on the interaction between H1 and ectopic H3 tails (Fig. 1A, lane 3; data not shown). Intriguingly, however, the ability of H1 to interact with the H3 tails was compromised upon K27 mutation which abolished cellular K27 methylation (lane 4), suggesting the importance of H3K27 methylation for H1-H3 tail interaction.

Next, we sought to examine whether the observed interaction is selective for any specific H1 subtypes and is dependent on mono-, di- or tri-methylation state of H3K27. Since we were mainly interested in the role of canonical, replication-dependent somatic linker histones, we decided to focus on H1.1-H1.5 subtypes in this study. Due to high sequence homology among human H1 subtypes, mass spectrometry analysis to distinguish H1 subtypes in H3 tail-associated factors turned out to be difficult, as in the case of our previous report^25^. For this reason, Flag-tagged somatic H1 subtypes H1.1-H1.5 were prepared by employing a bacterial expression system and utilized for the binding experiments. The C-terminally biotinylated peptides corresponding to the N-terminal H3 tail (aa 21–44) either unmodified (K27me0), monomethylated (K27me1), dimethylated (K27me2), or trimethylated (K27me3) at K27 were immobilized on streptavidin-coated wells, and monitored the binding for recombinant H1.1-H1.5 by colorimetric assays. Somewhat surprisingly, our results showed that H1.2 preferentially interacted with biotinylated H3K27me3 tail peptides, whereas other H1 subtypes were unable to display any binding preference for H3K27me3 tail peptides, in a binding buffer containing 200 mM KCl and poly(dA-dT) (Fig. 1B). In additional binding experiments with GST-H1.2 attached to glutathione beads under increasing KCl concentrations, the interaction of FITC-conjugated H3K27me3 peptides with GST-H1.2 was maximal at the lowest KCl concentration (100 mM) and decreased upon increasing KCl concentrations from 100 to 400 mM (Fig. S1A). On the other hand, H3K27me0, H3K27me1 and H3K27me2 peptides showed markedly lower H1.2 binding at 100 mM KCl, and displayed nearly 3-fold weaker interaction at elevated KCl concentrations (Fig. S1A).

To examine the observed interaction further, nucleosomes were reconstituted from the biotinylated 601 nucleosome positioning sequence and histone octamers containing semisynthetic K27-unmethylated or K27-trimethylated H3, and immobilized onto streptavidin-conjugated magnetic beads. After incubation with Flag tagged H1.1-H1.5 proteins in a binding buffer containing 150 mM KCl, immobilized nucleosomes were spun down and subjected to Western blot analysis with anti-Flag antibody. These binding assays clearly demonstrated that H1.2 binds to H3K27me3 nucleosomes with an affinity much higher than to H3K27me0 nucleosomes (Fig. 1C, lanes 1–4). The magnitude of the H3K27me3 effects on H1.2 binding to nucleosomes was even greater in 250 mM KCl (lanes 5–7). The lack of an effect of H3K27me3 on the binding of H1.1 and H1.3-H1.5 indicate that enhanced binding of H1.2 is not due to changes in linker DNA accessibility.

The preferential interaction of H1.2 with H3K27me3 nucleosomes was also confirmed by our quantitative binding experiments in which H3K27me0 and H3K27me3nucleosomes were immobilized on streptavidin-coated wells and the binding of recombinant H1.1-H1.5 was monitored by using colorimetric detection system (Fig. S1B). In additional binding experiments with truncated versions of H1.2, H1.2 deleted of N-terminal tail (amino acids 1–34) still retained high affinity for immobilized H3K27me3 nucleosomes (Fig. 1D, lane 9). H1.2 deletion mutants lacking amino acids 181–213 or 146–213 also showed the direct interaction with H3K27me3 nucleosomes (lanes 10 and 11), but further deletion of the remainder (amino acids 110–145) of the C-terminal tail failed to generate detectable interaction (lanes 8 and 12).

As the H1 subtypes show a high degree of sequence conservation^3^,^5^, the observed interaction of H1.2 C-terminal tail stretch consisting of amino acids 110–145 with H3K27me3 nucleosome might be dependent on a small number of amino acid residues. When comparing amino acid sequences in the region between residues 110 and 145 of the five somatic H1 subtypes, we found the three unique amino acids V120, T126 and V132 that could be critical for H1.2 interaction with H3K27me3 nucleosomes (Fig. S1C, left panel). In the first set of binding experiments in 250 mM KCl buffer, individual mutations of V120, T126 and V132 showed no apparent effects on H1.2 binding to nucleosomes (Fig. S1C, right panel). However, simultaneous mutations of the three residues significantly incapacitated H1.2 from binding to H3K27me3 nucleosomes (Fig. S1C, right panel). In concordance with these results, additional binding assays employing FITC-labeled nucleosomes and immobilized GST-H1.2 proteins at various salt concentrations demonstrated that H3K27me3 nucleosomes interacted with the triple mutant H1.2 much more weakly than wild type H1.2 in the salt concentrations ranging from 200 mM to 500 mM KCl (Fig. 1E). The importance of the three amino acids V120, T126 and V132 was also confirmed by the finding that their mutations caused a significant decrease in H1.2 binding to FITC-conjugated H3K27me3 peptides (Fig. S1A). To further assess the significance of V120, T126 and V132 with respect to H1.2-H3K27me3 nucleosome interaction, we introduced these amino acids in H1.4 by point mutations and performed *in vitro* binding assays. The H1.2 mimicking mutations of H1.4 led to a marked increase in the binding affinity for H3K27me3 nucleosomes (Fig. S2).

**Figure 2:**
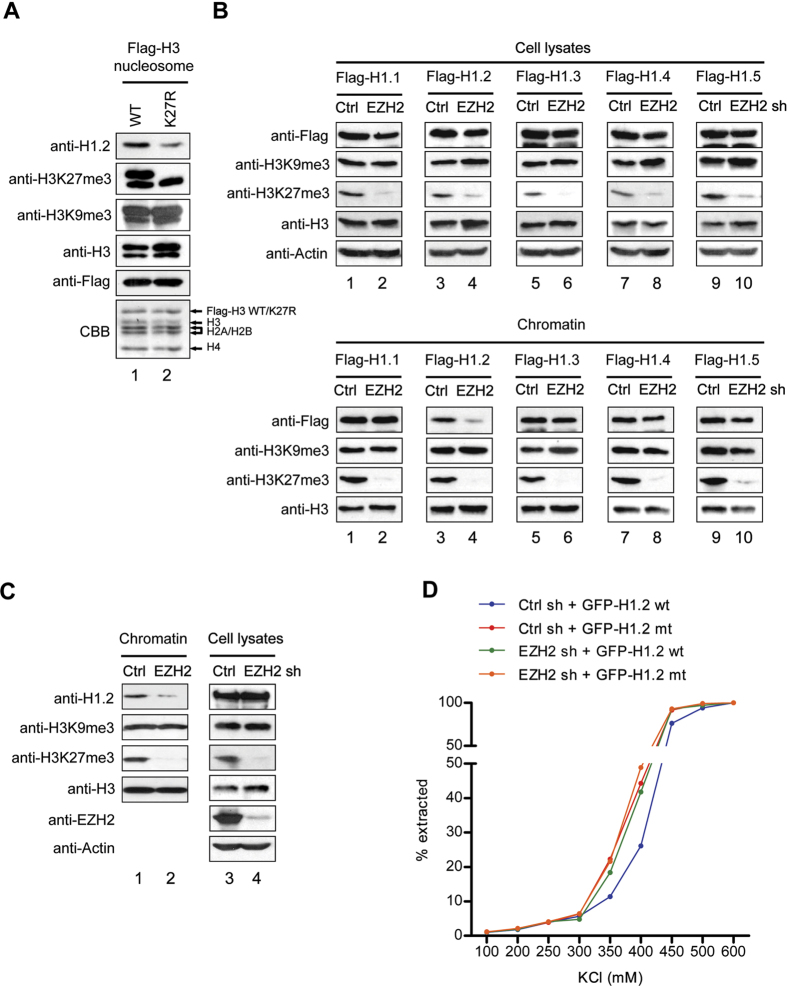
H1.2 interaction with H3K27me3 nucleosomes *in vivo*. (**A**) MCF7 cells were transfected with Flag-tagged wild type (WT) or K27-mutated (K27R) H3, and mononucleosomes were prepared by MNase digestion. Mononucleosomes containing ectopic H3 were immunoprecipitated from total mononucleosomes with Flag antibody, and analyzed by Western blot with the indicated antibodies. (**B**) EZH2-depleted MCF7 cells were transfected with expression vectors for Flag-H1 subtypes. Forty-eight hours post-transfection, whole cell extracts and chromatin fractions were prepared and subjected to Western blotting with the indicated antibodies. Random nontargeting shRNA-transfected MCF7 cells were used as controls (Ctrl). (**C**) Whole cell lysates and chromatin were prepared from control (Ctrl) and EZH2-depleted (EZH2) MCF7 cells and analyzed by Western blot as in (**A**). (**D**) Control (Ctrl) and EZH2-depleted (EZH2) MCF7 cells were transfected with expression constructs encoding GFP-H1.2 wild type (wt) and GFP-H1.2 V120/T126/V132 mutant (mt) for 48 h. Nuclei were isolated and resuspended in buffers containing increasing concentrations of KCl. The fluorescence intensity of extracted GFP-H1.2 was measured using a fluorescence microplate reader. Data shown are representative of three independent experiments.

In an attempt to confirm these observations *in vivo*, MCF7 breast cancer cells were transfected with plasmids expressing Flag-wild type or K27-mutated H3, and mononucleosomes containing ectopic H3 were purified following the procedure described^26^. We confirmed that similar levels (~60%) of ectopic wild type and mutant H3 proteins are present in the purified nucleosomes by Coomassie blue staining as well as anti-H3 Western blot (Fig. 2A). In our analysis using H1.2 antibody, we detected a stable association of H1.2 with the wild type H3 nucleosomes (Fig. 2A). However, the observed interaction was reduced about 2.5-fold in the H3K27-mutated nucleosomes, although the residual interaction was detectable due to the K27me3 of cellular H3 in the nucleosomes. To further investigate the impact of H3K27me3, we suppressed the expression of EZH2 which is mainly responsible for H3K27me3 in MCF7 cells^24^,^27^ and evaluated its effects on chromatin binding properties of H1 subtypes. Relative to non-targeting control shRNA, shRNA directed against EZH2 efficiently depleted EZH2 and almost completely abrogated H3K27me3 (Fig. 2B,C). This decrease in EZH2-mediated H3K27me3 generated a significant reduction of H1.2 binding to chromatin, but had little to no effect on the binding of other H1 subtypes, as confirmed by Western blot analysis of purified chromatin fractions (Fig. 2B,C). Another demonstration in support of the preferential interaction of H1.2 with H3K27me3-enriched chromatin came from the results obtained from salt extract experiments. This approach takes advantage of the fact that higher salt concentrations are required to extract H1.2 proteins that are more tightly bound to chromatin in nuclei. Both wild type and V120/T126/V132-mutated H1.2 proteins were minimally solubilized at 300 mM or lower salt concentrations, and increasing the salt concentration of the extraction buffer up to 450 mM supported the dissociation of about 90% of mutant H1.2 from chromatin (Fig. 2D). By comparison, wild type H1.2 was much less dissociated from chromatin under the same extraction conditions. In addition, EZH2 knockdown generated a significant increase in soluble nuclear H1.2, indicating that H1.2 proteins are more tightly bound to chromatin in an H3K27me3-dependent manner (Fig. 2D).

### H3K27me3 is important for H1.2-mediated chromatin compaction and transcriptional repression

**Figure 3:**
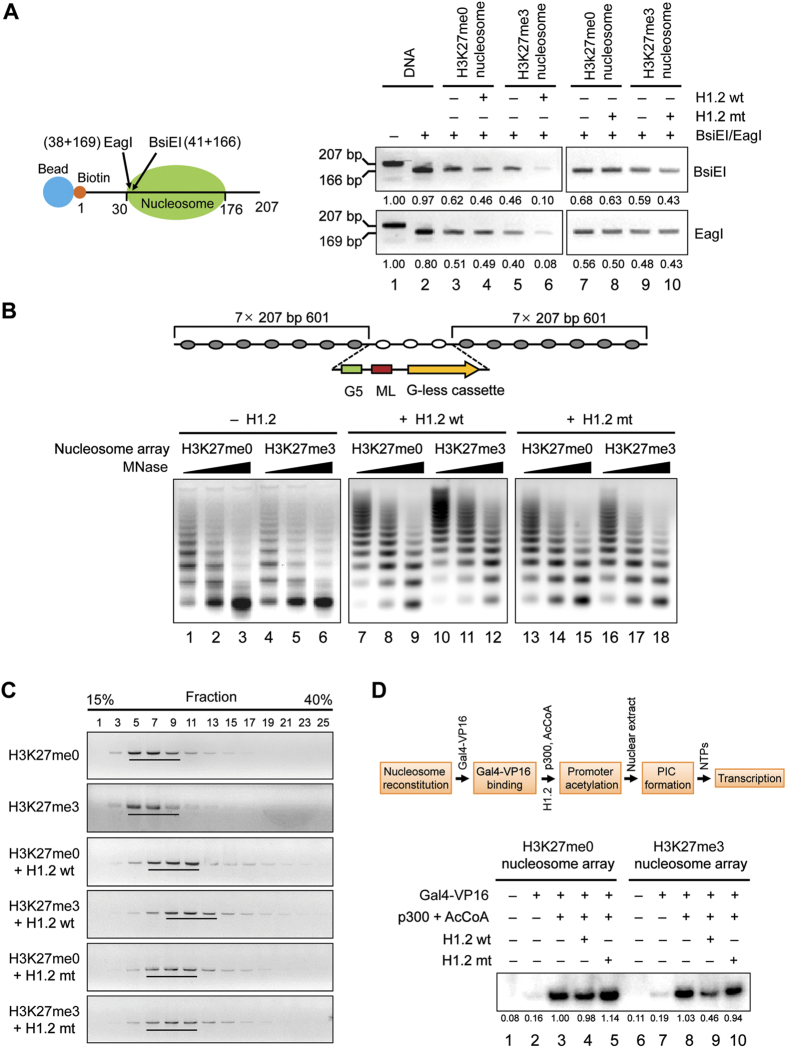
Stimulation of H1.2-mediated chromatin compaction by H3K27me3. (**A**) H3K27me0 or H3K27me3 601 nucleosomes were immobilized on streptavidin agarose beads, incubated with H1.2 wild type (wt) or H1.2 V120/T126/V132 mutant (mt), and digested with BsiEI and EagI. After washing and Proteinase K digestion, DNA fragments released from beads were ethanol precipitated and analyzed by 2.5% agarose gel electrophoresis. The left panel shows the schematic illustration of 207 bp 601 nucleosome positioning sequence. The green oval and arrows indicate nucleosome position and restriction enzyme cleavage sites, respectively. Data shown are representative of three independent experiments. Band intensities were quantified and normalized relative to DNA reactions. (**B**) Nucleosome arrays containing H3K27me0 or H3K27me3 were reconstituted on G5ML601 array templates, incubated with wild type (wt) or mutant (mt) H1.2, and digested with increasing concentrations of MNase for 10 min. The digestion products were run on 1% native agarose gels, and stained with ethidium bromide. Data show a representative result from three independent experiments. (**C**) Nucleosome arrays containing H3K27me0 or H3K27me3 were incubated with H1.2 wild type (wt) or mutant (mt), and separated by 15–40% glycerol gradient high speed centrifugation. Aliquots of every other fraction from the gradient were analyzed by 1% DNA agarose gel stained with ethidium bromide staining. (**D**) Nucleosome arrays containing H3K27me0 or H3K27me3 were transcribed with Gal4-VP16, p300 and AcCoA in the presence of H1.2 wild type (wt) or H1.2 V120/T126/V132 mutant (mt) as indicated above the panel. The results shown are representative of three independent experiments. Data were quantified by Image Gauge.

Because H3 N-terminal tails are well exposed outside of the nucleosome at the region where the DNA enters and exits the nucleosome^2^, it is possible that H3K27me3-facilitated H1.2 binding affects nucleosome stability. To explore this possibility, we reconstituted mononucleosomes on a 5′-biotinylated 207 bp derivative of the 601 nucleosome positioning sequence, and performed restriction enzyme accessibility assays. Nucleosomes were incubated with H1.2, immobilized on streptavidin-conjugated magnetic beads, and monitored the accessibility of nucleosomal DNA by digestion with two restriction enzymes, BsiEI and EagI, whose recognition sites are located near the 5′ end of the nucleosome (Fig. 3A, left panel). Since nucleosomes will be released from the beads by digestion with BsiEI and EagI, we compared the amounts of nucleosomes in the supernatant. In the absence of H1.2, the 601 positioning sequence was not very resistant to activities of BsiEI and EagI restriction enzymes, which produce 166 bp and 169 bp DNA fragments, respectively, in both H3K27me0 and H3K27me3 nucleosomes (Fig. 3A, right panel). Our results are consistent with those of earlier studies^28^,^29^ and indicate that the entry and exit points of nucleosomal DNA are more accessible for restriction enzyme digestion compared to other parts of nucleosomal DNA. When H3K27me3 nucleosomes were incubated with wild type H1.2 at a molar ratio of about one H1.2 per nucleosome, the accessibilities of the two restriction enzymes to their target sites dropped significantly (Fig. 3A, right panel). On the contrary, the incubation of H3K27me0 nucleosomes with H1.2 causes essentially no change in the accessibility of nucleosomal DNA target sites (right panel). The addition of H3K27me3 binding-deficient H1.2 mutant to nucleosomes also left the accessibility unchanged (right panel), further consolidating the results.

As an extension of the above-described studies using mononucleosomes, it was also important to analyze the impact of H3K27me3-facilitated H1.2 binding on chromatin accessibility. For this objective, nucleosome arrays were reconstituted with H3K27me0 or H3K27me3 histone octamers onto the G5ML-601 array DNA template containing the adenovirus major late promoter, G-less cassette, Gal4 binding sites, and 14 copies of a 207 bp 601 nucleosome positioning sequence (Fig. 3B, upper panel). Digestion of H3K27me0 and H3K27me3 nucleosome arrays with low concentrations of micrococcal nuclease (MNase) produced a ladder of DNA fragments, whereas a high concentration of the enzyme gave rise mostly to mononucleosome-length DNA fragments (lower panel). When H3K27me3 nucleosome arrays were preincubated with H1.2 proteins and digested with MNase, the decreased accessibility to H3K27me3 nucleosome arrays was more evident upon inclusion of wild type H1.2, compared to mutant H1.2 (lower panel). However, MNase digestion of H3K27me0 nucleosome arrays in the presence of H1.2 generated MNase digestion patterns similar to those of H3K27me3 nucleosome arrays incubated with mutant H1.2 (lower panel).

Although the observed resistance to MNase digestion does not necessarily reflect the role of H1.2 in H3K27me3-induced chromatin compaction, these results suggest H1.2 binding to H3K27me3 nucleosomes might have effects on local chromatin structure. To examine this possibility, we analyzed the sedimentation velocity of nucleosomes arrays by ultracentrifugation in a linear 15%–40% glycerol gradient. In these analyses, H3K27me0 and H3K27me3 nucleosome arrays generated equivalent levels of sedimentation in the absence of H1.2 (Fig. 3C). A slight enhancement of sedimentation was observed in a parallel analysis with mutant H1.2. Importantly, a sedimentation analysis using wild type H1.2 revealed a more distinct shift of H3K27me3 nucleosome arrays toward high molecular weight fractions in the gradient. These results, albeit indirect, strongly implicate H1.2 in H3K27me3-induced chromatin compaction and transcription inhibition.

Because a role for linker histone H1 as a transcription repressor is well established^7^,^30^,^31^, we sought to determine whether H3K27me3 influences the ability of H1.2 to inhibit transcription. To accomplish this, we adopted a nucleosome array-based transcription assay system and performed *in vitro* transcription experiments in which G5ML 601 nucleosome array templates containing H3K27me0 or H3K27me3 were transcribed with the activator Gal4-VP16 and cofactor p300 (Fig. 3D, left panel). When nucleosome arrays were transcribed with Gal4-VP16 and p300, high levels of transcription were obtained from both H3K27me0 and H3K27me3 nucleosome arrays (Fig. 3D, right panel, lanes 3 and 8). In assessing the effects of H1.2 on transcription, we found that adding H1.2 to H3K27me0 nucleosome arrays at a molar ratio of one H1.2 per nucleosome did not alter the levels of transcription (lane 4). However, if H1.2 was added to transcription reactions with H3K27me3 nucleosome arrays, distinct repression could be observed (lane 9), indicating that H1.2 represses transcription in an H3K27me3-dependent manner. Moreover, the fact that adding H3K27me3 binding-deficient H1.2 mutant to H3K27me3 nucleosome arrays minimally affected transcription (lane 10) strongly argues that the observed transrepression depends on the ability of H1.2 to recognize H3K27me3 marks in nucleosome array templates.

### H1.2 and EZH2 act cooperatively to silence growth regulatory genes in cancer cells

**Figure 4:**
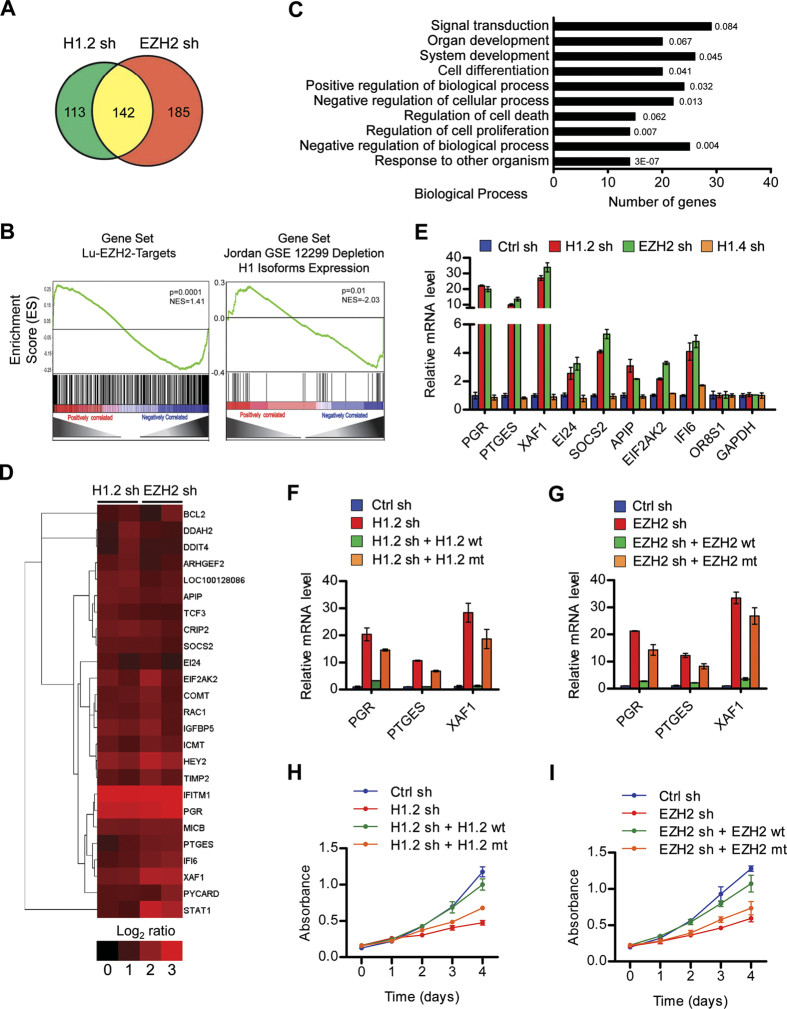
Transcriptional silencing of growth suppressive genes by H1.2 and EZH2. (**A**) MCF7 cells were depleted of H1.2 or EZH2 and subjected to microarray analysis. Venn diagrams show 142 genes that are commonly activated more than two fold in response to H1.2 and EZH2 knockdown in two independent analyses. (**B**) GSEA showed that genes regulated by H1.2 in MCF7 cells were similar to previously identified target genes of EZH2 (left panel). Normalized expression values for H1.2 shRNA and corresponding controls were used to rank the enrichment of genes in molecular signature database of Broad Institute using GSEA algorithm. A similar GSEA of EZH2 target genes also showed the enrichment of previously identified target genes of H1.2 (right panel). (**C**) Gene ontology analysis of the common targets of H1.2 and EZH2 using DAVID bioinformatics resources (http://david.abcc.ncifcrf.gov). (**D**) Heat map generated by TreeView analysis of the growth regulatory genes that were commonly upregulated upon knockdown of H1.2 and EZH2. (**E**) Microarray data were validated by qRT-PCR using primers specific for the 8 genes that were upregulated and the 2 genes that were unaffected following H1.2, EZH2 knockdown. Also included in the qRT-PCR analysis was mRNA extracted from H1.4-depleted MCF7 cells. Primer sequences are listed in Table S2. The values are expressed as fold changes from the mRNA levels in undepleted control cells. Data represent the means ± SD of three independent experiments. (**F**) H1.2-depleted MCF7 cells were infected with lentiviruses expressing wild type (wt) or V120/T126/V132-mutated (mt) RNAi-resistant H1.2, and relative mRNA levels of the target genes were quantified by qRT-PCR. (**G**) Wild type (wt) or H689-mutated (mt) RNAi-resistant EZH2 was expressed in EZH2-depleted MCF7 cells, and target gene expression was determined by qRT-PCR as in (**F**). Data in (**F,G**) represent the means ± SD of three independent experiments. (**H**) H1.2-depleted MCF7 cells were infected with wild type or mutant RNAi-resistant H1.2 as in (**F**), and changes in cell proliferation rates were measured by the MTT colorimetric assay. (**I**) EZH2-depleted MCF7 cells were complemented with wild type or mutant EZH2 as in (**G**), and MTT proliferation assays were carried over a period of 4 days. Data in (**H**) and (**I**) represent the means ± SD of three independent experiments.

The expression and activity of EZH2 are higher in numerous human cancers, and a connection between aberrant H3K27me3 and oncogenesis has been described^23^,^24^,^32^. We therefore proceeded to study the hypothesis that the above-described interaction of H1.2 with H3K27me3 nucleosomes alters specific gene expression and promotes tumorigenesis. Our Western blot analysis of cell lysates revealed the global levels of EZH2-mediated H3K27me3 and H1.2 much higher in MCF7 breast, LD611 bladder and LNCaP prostate cancer cells than in their nontransformed cells (MCF10-2A, LD419, and MLC) (Fig. S3A). To functionally investigate the observed changes in cancer cells, we purified total RNA from MCF7 cells expressing shRNAs against H1.2 and EZH2, and conducted microarray analyses using the Illumina humanHT-12 v4 Expression BeadChip arrays. With a fold change cutoff of >2 and stringent P <0.001, our analysis revealed that the expression of 255 and 327 genes was increased in response to knockdown of H1.2 and EZH2, respectively (Fig. 4A, Table S1). When the two gene lists were compared, 142 genes were found to be commonly activated after H1.2 and EZH2 knockdown, a functional link that had not been previously noted (Fig. 4A). A gene set enrichment analysis (GSEA) analysis of our microarray data with publicly available molecular signature datasets indicated that a statistically significant number (p < 0.0001 with a false discovery rate < 0.005) of the genes whose expression was altered in H1.2-depleted cells were EZH2 targets (Fig. 4B, Table S5). A similar GSEA of EZH2 microarray data also showed a statistically significant enrichment of the H1.2 targets identified in a previous study^9^ using the breast cancer cell line T47D (Fig. 4B, Table S5). In further analysis of our microarray data, a significant activation was observed for the genes involved in the control of cell death and proliferation (Fig. 4C,D). As an experiment to confirm the microarray results, quantitative RT-PCR (qRT-PCR) analysis of 8 putative target genes showed that H1.2 and EZH2 knockdown caused 3- to 30-fold increases in their mRNA levels (Fig. 4E). The observation that these genes are not affected by knockdown of another subtype H1.4 indicates that the putative target genes are selectively regulated by H1.2 (Fig. 4E). Moreover, the expression of wild type H1.2, but not H3K27me3 binding-deficient H1.2 mutant, resulted in, albeit to a varying extent, lower expression of the putative target genes in H1.2-depleted cells (Fig. 4F, Fig. S3B,D). Analogously, the ectopic expression of wild type EZH2 restored the inactive states of the candidate target genes in EZH2-depleted cells, but the enzymatically dead EZH2 H689A mutant^33^ had no discernible effect (Fig. 4G, Fig. S3C,E).

Given the demonstrated effects of H1.2 and EZH2 knockdown on growth-controlling genes, we also examined the cooperative roles of H1.2 and EZH2 with respect to cell proliferation. Our MTT assays over a 4-day time course showed that individual knockdown of H1.2 and EZH2 in MCF7, LD611 and LNCaP cancer cells gradually decreased cell proliferation rates (Fig. 4H,I, Fig. S3F,G). Also in checking the rescue potential of ectopic H1.2, wild type H1.2 fully restored the growth rate of the cancer cells depleted of H1.2, whereas H3K27me3 binding-deficient H1.2 was much less efficient in restoring the growth rates (Fig. 4H, Fig. S3F). Essentially identical results were obtained with EZH2-depleted cells (Fig. 4I, Fig. S3G). Thus, the expression of wild type, but not enzymatically inactive EZH2 mutant, EZH2 rescued cell proliferation rates. These experiments again confirm the specificity of our RNAi experiments and, taken together with our gene expression analyses, unequivocally demonstrate the direct requirements of H1.2 and EZH2 for transcriptional silencing of growth suppressive genes.

### H1.2 and EZH2-mediated H3K27me3 display similar localization patterns at target genes

**Figure 5:**
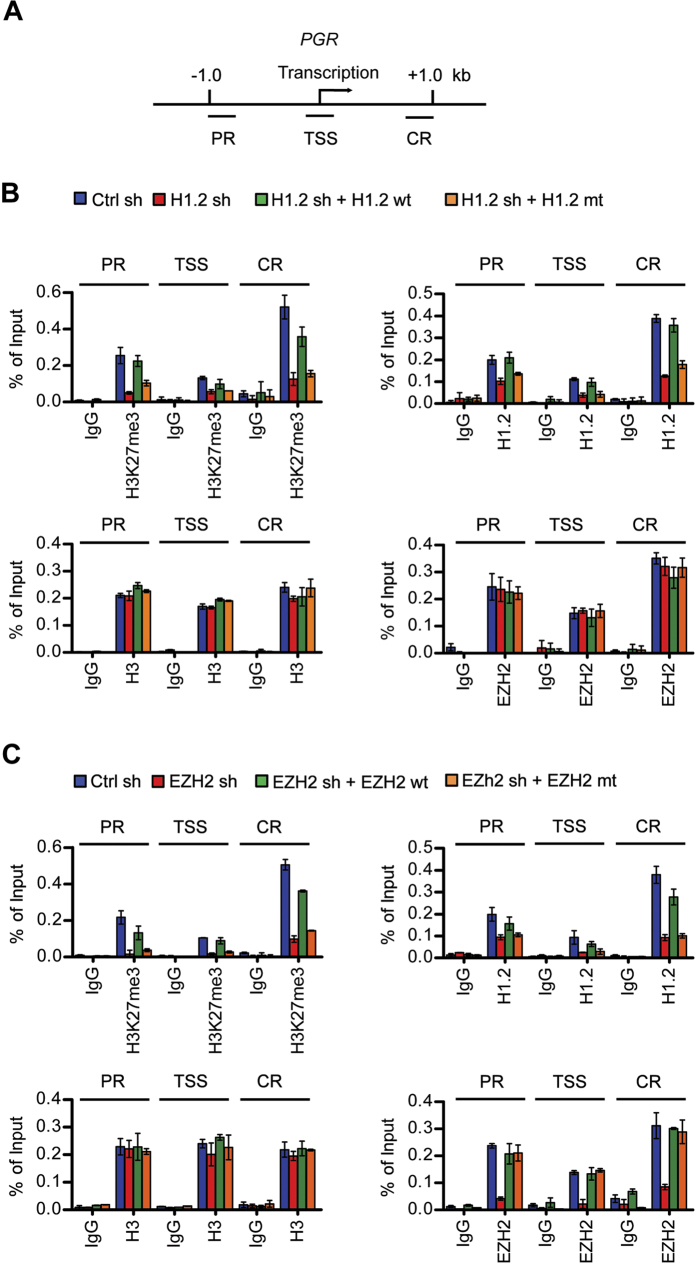
Colocalization of H1.2 and EZH2-mediated H3K27me3 at target genes. (**A**) Approximate locations of three amplicons at the *PGR* locus used in the ChIP assays are shown. (**B**) ChIP assays were performed in mock-depleted (Ctrl sh) and H1.2-depleted (H1.2 sh) MCF7 cells using antibodies against H3K27me3, H1.2, EZH2 and H3 as indicated. To rescue the effects of H1.2 knockdown, H1.2-depleted cells were transfected with wild type (wt) or V120/T126/V132-mutated (mt) RNAi-resistant H1.2. Precipitation efficiencies relative to non-enriched input samples were determined for the three locations across the *PGR* locus by qPCR with primers depicted in (**A**) and listed in Table S3. Percent input is determined as the amount of immunoprecipitated DNA relative to input DNA. (**C**) ChIP assays were carried out as in (**B**), but using EZH2-depleted MCF7 cells. For EZH2 rescue experiments, wild type (wt) or H689A-mutated (mt) RNAi-resistant EZH2 was expressed. Data in (**B,C**) represent the means ± SD of three independent experiments.

Overall, our microarray results establish the functional interaction between H1.2 and EZH2 leading to transcriptional inactivation of particular sets of genes in cancer cells. However, it is not clear whether the observed effects of H1.2 and EZH2 reflect their targeted recruitments and activities. To check this possibility, we conducted chromatin immunoprecipitation (ChIP) assays employing chromatin isolated from control cells and cells depleted of H1.2 or EZH2. The precipitated DNA was amplified by quantitative PCR (qPCR) using primers specific for the promoter region (PR), transcription start site (TSS) and coding region (CR) of the target genes, as summarized in Fig. 5A and Fig. S4A. In mock-depleted cells, H3K27me3 levels were high at the promoter and coding regions, although the enrichment patterns were slightly different among the target genes (Fig. 5B,C, Fig. S4B-G). H1.2 occupancy patterns across the target genes were similar to those observed for H3K27me3, implicating H3K27me3 as the major recruitment signal for H1.2 (Fig. 5B,C, Fig. S4B-G). H3K27me3 levels were reduced at the target genes after EZH2 knockdown, and such changes diminished the localization of H1.2 at target loci (Fig. 5C, Fig. S4C,E). These results were validated by rescue experiments demonstrating that the ectopic expression of RNAi-resistant wild-type, but not H689A mutant, EZH2 restored H3K27me3 and H1.2 to levels comparable to control cells (Fig. 5C, Fig. S4C,E). No detectable effects of H1.2 knockdown on EZH2 distribution across the target genes indicate that H1.2 is dispensable for the initial recruitment and stable occupancy of EZH2 at the target genes (Fig. 5B, Fig. S4B,D). Interestingly, however, H1.2 knockdown significantly decreased the levels of H3K27me3 at the target genes. These findings are in full agreement with a recent report^34^ and support the idea that higher H1.2 content at target loci leads to more compact chromatin structure and generates the ideal substrate for EZH2 enzymatic activity. To further confirm these results, we expressed Flag-H1.4 to the level similar to that of Flag-H1.2 in H1.2-depleted cells (Fig. S5B), and repeated ChIP analysis at the target genes. These assays detected no obvious effects of EZH2 and H1.2 knockdown on the localization of ectopic H1.4 at target loci (Fig. S5D), and taken together with our qRT-PCR analysis (Fig. S5C), demonstrate a selective contribution of EZH2 to H1.2 occupancy and transrepression of the target genes.

### H1.2 reduces chromatin aNccessibility around target loci in an EZH2-dependent manner

**Figure 6:**
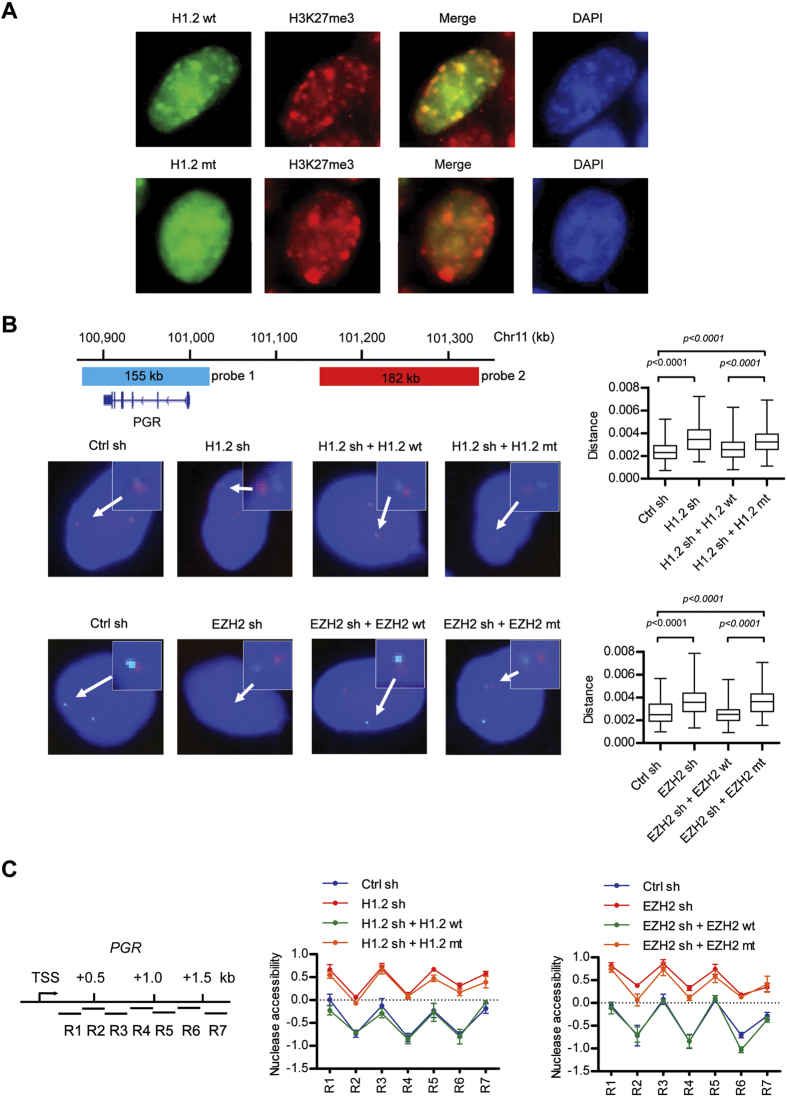
Requirement of H1.2 and EZH2 for higher order chromatin organization around target loci. (**A**) MCF7 cells were transfected with expression constructs encoding GFP-wild type (wt) H1.2 and GFP-V120/T126/V132-mutated (mt) H1.2, and were immunostained for GFP-H1.2 (green), H3K27me3 (red) and DAPI (blue). Image overlays show co-localization of H3K27me3 with wild type H1.2, but not with mutant H1.2, in several nuclear areas. Representative images of transfected cells are shown. (**B**) A diagram of the chromosome 11 region shows the positions of the 5-Fluorescein-labled *PGR* probe (probe 1) and 5-Carboxyl-rhodamine-labled upstream probe (probe 2) used in the FISH analysis. Shown on the left are representative images from FISH with the probe pairs in nuclei of control cells, EZH2/H1.2-depleted cells, and EHZ2/H1.2-depleted cells expressing wild type or mutant EZH2/H1.2. One hundred nuclei were used to measure interprobe distances for each condition, and the statistical significance of differences between the indicated two groups was measured by the Mann-Whitney U test. The right panel graphically shows the distribution of interprobe distances in the indicated cells. (**C**) The accessibility of the *PGR* downstream nucleosomes in control and EZH2-depleted MCF7 cells was determined by the nuclease protection assay at the seven amplicons covering 2-kb downstream of the TSS. The rescue effects of wild type or mutant EZH2/H1.2 in EZH2-/H1.2-depleted cells were also analyzed. Similar results were obtained from three independent experiments.

Having established the importance of H3K27me3 in H1.2 localization and function, we next wished to determine whether the observed recruitment of H1.2 to target loci affects chromatin structure. To address this issue, we first expressed H1.2 fused to green fluorescent protein (GFP) in MCF7 cells and examined its nuclear localization by fluorescence microscopy. GFP signals for wild type H1.2 were primarily detected in the regions that were heterochromatin-dense, as indicated by the stronger staining with DAPI (Fig. S6A). By contrast, cells expressing GFP-mutant H1.2 showed the GFP fluorescence patterns somewhat different from the patterns obtained using DAPI (Fig. S6A). We also found that the H3K27me3 antibody preferentially stained a nuclear region that co-localizes with the wild type H1.2 (Fig. 6A). However, the observed localization of H1.2 to H3K27me3-enriched heterochromatin regions was significantly compromised by mutations of the three H1.2-specific amino acids, which are critical for H1.2 binding on H3K27me3 nucleosomes (Fig. 6A). These results indicate that H3K27me3 is necessary for H1.2 to be detected at condensed heterochromatic regions.

We then performed fluorescence *in situ* hybridization (FISH) on MCF7 cells and tested whether H3K27me3 could direct H1.2 to drive chromatin reorganization at the target genes. For these studies, we used the probe 1 that is specific for *PGR* locus and the probe 2 that spans approximately 182 kb of sequence upstream of the *PGR* locus (Fig. 6B, upper left panel). Our dual color FISH analysis using these two probes verified that the *PGR* locus and the upstream region are in close proximity in the majority of cells (middle and lower panels, Ctrl sh). Similar FISH analyses with cells depleted of H1.2 or EZH2 detected larger spatial distance between the *PGR* gene and upstream region (H1.2 sh and EZH2 sh), which supports the architectural reorganization of chromatin across these regions. Expectedly, however, ectopically expressing wild type, but not mutant, H1.2 and EZH2 proteins restored the original distance between the two probes (H1.2 sh + H1.2 wt, H1.2 sh + H1.2 mt, EZH2 sh + EZH2 wt, and EZH2 sh + EZH2 mt). Moreover, and further indicative of highly specific functions of H1.2 and EZH2 at the target genes, FISH using non-target gene probes failed to show the effects of knockdown and expression of H1.2 and EZH2 (Fig. S6B). Considered together, these results lead us to conclude that EZH2-mediated H3K27me3 plays an especially important role in recruiting H1.2 to the target genes and mediating the structural and spatial reorganization of chromatin.

As another experiment to prove the importance of H1.2 and EZH2 in the regulation of chromatin architecture around the target genes, we measured the sensitivity of the 5′ coding region of the *PGR* gene to MNase digestion. Soluble chromatin was isolated from mock-depleted or H1.2/EZH2-depleted MCF7 cells, and digested down to mononucleosomes by MNase. Nucleosomal DNA was purified and amplified by qPCR using a series of overlapping primer pairs covering a 2-kb window immediately downstream of the TSS (Fig. 6C, left panel). This region was chosen for testing because it contains high levels of H1.2 and H3K27me3, as identified in our ChIP analyses (Fig. 5). Quantification and normalization of MNase digestion results showed that nucleosomes residing *PGR* coding region are more readily digested by MNase, when purified from H1.2-depleted cells (Fig. 6C, middle panel). Similar results were obtained with nucleosomes purified from EZH2-depleted cells (right panel). As further validation of these results, our rescue experiments demonstrated that the expression of wild type H1.2 and EZH2, but not their mutant counterparts, largely overrides the elevated nucleosome accessibility arising from H1.2 and EZH2 knockdown (middle and right panels). We repeated these experiments for the three other target genes *PTGES*, *XAF1*, *OR8S1* exhibiting higher accumulation of EZH2-mediated H3K27me3 and H1.2 at the promoter region; similar results were obtained from promoter chromatin (Fig. S6C-E). Overall, these results establish H1.2 functions in the formation and maintenance of local chromatin domains in a less accessible state and further suggest that the observed capacity of H1.2 is apparently dependent upon EZH2-mediated H3K27me3.

## Discussion

Linker histone H1 has traditionally been considered a structural component of the nucleosome, allowing the high degree of chromatin compaction and inaccessibility. However, more recent studies employing gene expression microarray and knockdown technologies suggest that linker histone H1 subtypes differ in functional characteristics and are engaged in the regulation of specific gene expression^9^,^26^,^35^. If H1 subtypes contribute to gene-specific transcription outcomes, then they should be enriched at distinct loci in a specific manner. Putting together the findings from *in vitro* and *in vivo* experimental approaches used in this study, we would like to suggest that H3K27me3 is the primary determinant of H1.2 localization at specific genes. We provide several lines of evidence to support this conclusion. First, H1.2 selectively interacts with H3 N-terminal tails bearing K27me3, and this interaction is required for efficient binding of H1.2 to nucleosomes. Second, H1.2 can localize at several growth suppressive genes in a manner dependent on EZH2 which is primarily responsible for H3K27me3 in breast, bladder, and prostate cancer cells. Third, there is a strong overlap between H1.2 localization and H3K27me3 enrichment at the target genes, especially at their proximal promoter and coding regions. Fourth, EZH2-dependent generation of H3K27me3 mark is necessary for H1.2-induced chromatin compaction observed in cancer cells. Fifth, the three amino acids V120, T126 and V132 in the C-terminal tail are unique for H1.2, and mutation of these residues yields H1.2 mutant that is unable to interact with H3K27me3 nucleosomes. Together, our data establish the C-terminal tail extension of H1.2 as an H3K27me3 recognition module and provide unprecedented insight into the biochemical mechanism underlying H1.2 localization at particular chromatin domains.

In relation to these results, a previous report showed the interaction of *Caenorhabditis elegans* H1 with H3K27me3 but, unlike the present study, used HIS-24, a homolog of human H1.1 and total worm lysates for binding assays^36^. Because H1 subtypes can be co-purified with multiple other factors^26^, HIS-24 may indirectly associate with H3K27me3. As an alternative explanation, HIS-24 modification might modulate its interaction with H3K27me3^37^. Although not clearly stated which H1 subtypes were employed, a recent study also demonstrated that H1 can bind the C-terminal tail of H2A in a nucleosome context^38^. These data hint at the possibility that other histone tails and their modifications are also involved in recruiting H1 to genomic targets. Therefore, a better understanding of differential roles of H1 subtypes in gene regulation will require studies on how specific histone marks are established and how they contribute to H1 localization at distinct chromatin regions.

Since H1 is known as a general regulator of chromatin organization, an expected outcome of H1.2 being incubated with nucleosome arrays would be inhibitory effect on transcription reactions. In contrast to these expectations, we failed to see any significant changes in transcription after adding H1.2 to unmodified nucleosome arrays at a nucleosome:H1.2 ratio of 1:1. However, if higher amounts of H1.2 were used, some repression could be observed (data not shown), indicating that H1.2, if used at non-physiological concentrations, may reorganize overall chromatin structure and leads to non-specific repression. On the other hand, H1.2 is able to abrogate transcription from H3K27me3 nucleosome arrays at a ratio of one H1.2 per nucleosome, consistent with its ability to bind H3K27me3 nucleosomes. Because H1 proteins interact dynamically with chromatin, continuously exchanging between nucleosomes^39^,^40^, the transient interaction of H1.2 with chromatin in part explains why H1.2 does not interfere with chromatin transcription with high efficiency in the absence of H3K27me3. In this regard, our data favor the mechanism in which H3K27me3 stabilizes H1.2 binding to nucleosomes and tightens chromatin organization to impede gene expression. It is also notable that natural core histones purified from cell nuclei were used to reconstitute nucleosome/chromatin in some previous H1 works, but these histone molecules contain prior cellular modifications including H3K27me3. Thus, H1 binding to nucleosomes without H3K27me3 reaction observed in these studies likely reflects the effects of pre-existing H3K27me3 of native histones.

An unsolved question in our study is how H1.2 plays such a specific role in H3K27me3-mediated gene silencing. The C-terminal tail of H1 appears to adopt a distinct secondary structure upon binding to nucleosomes for the continuous control of chromatin structure^41^,^42^,^43^. Thus it is possible that specific amino acids in the C-terminal tail domain of H1.2 furnish the architectural features necessary for the interaction with the N-terminal tail of H3K27me3. This is consistent with our observation that the mutations of the three unique amino acids V120, T126 and V132 of H1.2 disrupt H1.2 binding to H3K27me3 nucleosomes. It is also noteworthy that H1.2- and H3K27me3-mediated transcription repression observed in our defined *in vitro* system occurs independently of PRC1 complex. Hence, although H3K27me3 is known to recruit PRC1 for chromatin condensation and gene silencing^16^, our results demonstrate an intrinsic ability of H1.2 to coordinate with H3K27me3 in the absence of PRC1.

Misregulation of H1 expression and EZH2 function has been linked to tumorigenesis^23^,^24^,^32^,^44^,^45^. Consistent with this implication, our microarray analyses using cancer cells revealed a clear interplay of H1.2 and EZH2 in maintaining the inactive state of growth suppressive genes, as loss of EZH2 function resulted in the derepression of approximately half of the genes that are repressed by H1.2. These data provide strong evidence that H1.2 and EZH2 functionally interact and are required for maintaining the growth suppressive genes in an inactive state. Our ChIP assays on several target genes whose repression was found to be dependent upon H1.2 and EZH2 also show that H1.2 is recruited to target loci in an EZH2-dependent manner. These results are in full agreement with recent reports of H1.2 prevalence in repressed genes in breast cancer cells and oncogenic role for EZH2^13^,^46^,^47^, and offer a mechanistic explanation for the requirement of H1.2 during EZH2-mediated cancer development. Therefore, further characterization of cooperative activities of H1.2 and EZH2 has potential implications in terms of cancer treatment.

Interestingly, we also found that the ability of EZH2 to methylate target nucleosomes is dependent on chromatin structure; comparison of levels of H3K27me3 showed a dramatic decrease in H3K27me3 at the target genes after H1.2 knockdown that makes chromatin less compact. Consistent with our results, a recent study showed that the enzymatic activity of EZH2 in PRC2 complex is stimulated by chromatin condensation^34^. Since H1.2 binds to nucleosomes bearing H3K27me3, it is tempting to speculate that high levels of H3K27me3 via H1.2-induced compaction of chromatin generate a feedback loop capable of facilitating the recruitment and function of H1.2 at the target genes. Based on these results, we propose a model in which H1.2 binds to H3K27me3 nucleosomes, thereby promotes chromatin compaction, which in turn facilitates EZH2-mediated H3K27me3 in adjacent nucleosomes and enables H1.2 to direct the higher-order folding of chromatin. This positive feedback mechanism helps to explain, at least in part, the dynamic function of H1.2 and further emphasizes the need to decipher other regulatory networks governing gene-specific function for H1.2 and other H1 subtypes.

In addition to the transrepressive function of H1.2 shown here, our previous study using 293T cells linked H1.2 to transcriptional activation, especially at the level of elongation^26^. As essential components of H1.2-mediated transactivation, the Cul4A E3 ubiquitin ligase and PAF1 elongation complexes functionally cooperate with H1.2 in this process^26^. Another interesting feature of this transactivation process is that H1.2 recognizes the serine 2 phosphorylation of RNA polymerase II (RNAPII) for the timely recruitment of the Cul4A and PAF1 complexes to target genes^26^. This coactivation function of H1.2 is more consistent with a bridging function whereby H1.2 acts as an adapter between RNAPII and other components of the transcriptional apparatus. In this regard, there are sharp differences in the fundamental mechanisms of H1.2-driven transrepression versus transactivation. When EZH2 signaling pathway is dominant in cancer cells, H3K27me3 levels are high at growth regulatory genes, and H1.2 seems to mainly participate in mediating transcriptional inactivation. In cells displaying minimal EZH2 activity and normal phenotype, H3K27me3 levels are low, and H1.2 can be easily switched from a corepressor to a coactivator by interacting with the Cul4A and PAF1 complexes. Therefore, our present results, together with these previous observations, collectively provide a functional picture of H1.2, and suggest possible mechanisms to account for the bifunctional behavior of H1.2 in maintaining transcriptionally active or inactive states of chromatin at a molecular level.

## METHODS

### Cell Lines, Constructs and Antibodies

See Supplementary Information for details of cell lines, constructs and antibodies.

### Recombinant Proteins and H3 Tail Peptides

All the details regarding the preparation of recombinant histone proteins and histone tail peptides can be found in Supplementary Information.

### Reconstitution of Mononuclesome and Nucleosome arrays

Mononucleosome and nucleosome arrays were reconstituted by salt gradient dialysis and purified by glycerol gradient centrifugation, as described in Supplementary Information.

### RNA Interference

For shRNA-based gene knockdown, DNA oligonucleotides encoding shRNAs specific for H1.2 mRNA coding region (5′-CCGAAGAAGAGCGCTAAGAAA-3′), EZH2 mRNA 3′ UTR region (5′-GAAACAGCTGCCTTAGCTTCA-3′) and H1.4 mRNA coding region (5′-CCAAGAAGAGCGCCAAGAAGA-3′) were annealed and ligated into the lentiviral expression vector pLKO.1^26^,^31^. Lentivirus particles were generated in 293T cells by co-transfecting plasmids encoding VSV-G, NL-BH and the shRNAs. MCF7, LD611 and LNCaP cells were infected by the viruses, and were selected with puromycin for two weeks.

### H3 Tail Peptide and Nucleosome Binding Assays

Binding experiments using synthetic H3 peptides and reconstituted nucleosomes were performed as explained in detail in Supplementary Information.

### Cell-free Transcription

G5ML601 nucleosome arrays (100 ng) containing semisynthetic H3K27me0 or H3K27me3 were used for *in vitro* transcription assays. Typical transcription reactions contained Gal4-VP16 (15 ng), p300 (20 ng), AcCoA (10 μM) and/or H1.2 (100 ng). The radiolabeled transcript products from reactions were resolved on 5% polyacrylamide gels containing 7 M urea and detected by autoradiography.

### Restriction Enzyme Accessibility and MNase Digestion Assays

A detailed description of restriction enzyme and micrococcal nuclease digestion assays using reconstituted nucleosomes can be found in Supplementary Information.

### Gene Expression Microarray, qRT-PCR, and ChIP

Standard procedures were used, and details can be found in Supplementary Information.

### Chromatin Accessibility Assays

Assays were carried out as previously described^48^ with some modifications. Briefly, formaldehyde was added to MCF7 cell cultures at final 1% concentration, and cultures were incubated at 30 °C for 30 min with shaking. Cross-linking reactions were quenched by adding glycine to final concentration of 125 mM and incubating further at 30 °C for 5 min. Chromatin was isolated and digested with MNase to yield mononucleosomes in digestion buffer (20 mM HEPES, pH 7.4, 20 mM KCl, 2 mM CaCl_2_, 0.5 mM DTT, and 0.5 mM PMSF). After stopping digestion reactions by adding 1/10 vol of solution containing 250 mM EDTA and 5% SDS, samples were incubated at 65 °C for overnight to reverse the cross-linking. The DNA was cleared of protein/RNA by proteinase K/RNase H digestion and phenol extraction before ethanol precipitation. The enrichment of MNase digested DNA over 1.5 kb upstream or 2 kb downstream of TSS was determined by qPCR with the primers listed in Table S4. Protection signal changes were normalized to signals seen for *GAPDH* that does not show any altered expression after H1.2 and EZH2 knockdown, serving as a control region unchanged in MNase protection between experiments. The nuclease accessibility data were analyzed using EpiQ chromatin kit data analysis tool (www.bio-rad.com/epiq).

### Salt extractions and Fluorescence Microscopy

The salt extract experiments were performed as described^49^. In brief, control and EZH2-depleted MCF7 cells were transfected with wild type or mutant EZH2 fused to GFP. At 48 h post-transfection, nuclei were prepared from cells. Nuclei were resuspended at increasing salt concentrations and pelleted again, and the supernatant collected. Fluorescence light emitted by the collected samples was determined using a Plate Chameleon V plate reader. For fluorescence microscopy analysis, MCF7 cells were seeded onto coverslips and transfected with mammalian expression vectors for EGFP-wild type and mutant H1.2 using X-tremegene HP DNA transfection reagent per the manufacturer’s instructions (Roche). Cells were fixed in 4% formaldehyde, permeabilized, blocked, and incubated with the H3K27me3 antibodies for 12 h at 4 °C. Cells were stained with rhodamine-conjugated secondary antibodies (The Jackson Laboratory) and mounted onto glass slides. Microscopy was performed using a Zeiss Imager Z1 Apotome Microscope, and images were acquired with a Zeiss Axiocam digital camera.

### Fish

Cells were fixed with 4% paraformaldehyde for 10 min and permeabilized with 0.5% TritonX-100/PBS for 20 min. After washing with PBS, slides were incubated with 100 ug/ml RNase A for 1 h and 40 units/ml pepsin for 10 min at 37 °C, and dehydrated for 2 min each in 70%, 80% and 100% ethanol. Slides were denatured in 70% formamide for 5 min at 73 °C. DNA probes were prepared and labeled with rhodamine-dUTP and fluorescein-dUTP. Probes were denatured for 5 min at 73 °C and hybridized to the denatured slides for 24 h at 37 °C. Slides were washed for 5 min each in 50% formamide/2x SSC and 0.1% Tween 20/2x SSC at 42 °C, dehydrated for 2 min each in 70%, 80% and 100% ethanol, and mounted on glass slides. FISH signals were detected by a Zeiss imager Z1 Apotome Microscope. DNA probes used in these assays were the BAC RP11–599H8 (155 kb), RP11–942D19 (182 kb), BAC RP11–956H14 (185 kb) and RP11–96K4 (168 kb). Interprobe distances under each condition were calculated as described previously^50^. 100 cells (200 loci) were used to measure interprobe distances for each condition, and the statistical significance of differences between the indicated groups was measured by the Mann-Whitney U test.

### Cell Proliferation Assays

MCF7 cells were incubated with 0.5 mg/mL MTT (2-[4,5-dimethylthiazol-2-yl]-2,5-di-phenyltetrazolium bromide) (Sigma) for 2 h at 37 °C. The MTT formazan was dissolved in 200 μL of DMSO. The absorbance of the solution was quantified at 570 nm against 650 nm by a microplate reader (Bio-Rad).

### Chromatin Compaction Assays

Reconstituted nucleosome arrays were incubated with wild type or mutant H1.2 for 1 h, and were loaded on a 15%–40% glycerol gradient (25 mM HEPES-KOH, pH 7.6, 100 mM KCl, 1 mM EDTA, 2 mM MgCl_2_, 0.02% NP40, and 1 mM DTT). After centrifugation at 25,000 rpm for 5 h, and the fractions (150 μl) were analyzed by 1% agarose gel.

## Additional Information

**How to cite this article**: Kim, J.-M. *et al.* Linker histone H1.2 establishes chromatin compaction and gene silencing through recognition of H3K27me3. *Sci. Rep.* **5**, 16714; doi: 10.1038/srep16714 (2015).

## Supplementary Material

Supplementary Information

Supplementary Table S1

Supplementary Table S5
