# Supplementary material for: Linker histone H1.2 establishes chromatin compaction and gene silencing through recognition of H3K27me3
Source: Sci Rep. 2015 Nov 19;5:16714. doi: 10.1038/srep16714 (PMC4652225; doi:10.1038/srep16714)

SUPPLEMENTARY INFORMATION

Linker histone H1.2 establishes chromatin compaction and gene silencing through recognition of H3K27me3

Jin-Man Kim, Kyunghwan Kim, Vasu Punj, Gangning Liang, Tobias S. Ulmer, Wange Lu and Woojin An

SUPPLEMENTARY MATERIAL AND METHODS

Cell Lines, Constructs and Antibodies

MCF7 and LD611 cells were maintained in Dulbecco's modified Eagle's medium (DMEM) supplemented with 10% fetal bovine serum (FBS). LNCaP cells were cultured in RPMI culture media with 10% heat-inactivated FBS (Invitrogen). MCF10-2A cells were grown in a 1:1 mixture of DMEM and Ham's F12 supplemented with 5% horse serum. LD419 cells were maintained in McCoy's 5A medium containing 20% FBS. MLC cells were grown in T medium containing 10% FBS. Mammalian expression plasmids encoding wild type and mutant H3 tails were generated by subcloning the eight copies of corresponding cDNAs into pIRES as recently described[1]. For mammalian expression of wild type and mutant versions of H1.2, corresponding cDNAs were amplified by PCR and ligated into the correct reading frames of pIRES and pEGFP-C1 containing 5' Flag or GFP coding sequences. Further details of plasmid constructions are available upon request. Antibodies used in this study are as follows: H1.2 (ab4086), H3 (ab1791) and Biotin (ab1227) antibodies from Abcam, Actin (A1978) and Flag (A3165) antibodies from Sigma, EZH2 (C15410039) antibody from Diagenode, H3K9me3 (61013) antibody from Active Motif, H1 (SC-8616) from Santa Cruz Biotechnology, and H3K9me1 (07-450), H3K9me2 (07-441), H3K27me1 (07-448), H3K27me2 (07-452) and H3K27me3 (07-449) antibodies from Millipore.

Recombinant Proteins and H3 Tail Peptides

Recombinant core histones were expressed in E. coli Rosetta 2 (DE3) pLysS cells (Novagen), refolded to octamers, and purified as described previously[2]. H3 protein with trimethylated K27 was generated following the peptide ligation protocol and included in octamer preparation[3]. Bacterially expressed linker histones were purified by a three-step procedure using M2 affinity, CM-Sephadex C-25, and SP-HP chromatographies as recently described[4]. Unmodified and K27 mono-, di- and trimethylated H3 peptides corresponding to amino acids 21-44 of human H3 (ATKAARKSAPATGGVKKPHRYRPG) were synthesized by solid-phase Fmoc/tBu chemistry using an automated peptide synthesizer. The synthesized peptides were purified by RP-HPLC, and peptide purity was confirmed using ES-MS and amino acid analysis.

Reconstitution of Mononuclesome and Nucleosome arrays

For mononucleosome reconstitution, the 207 bp 601 sequence was PCR-amplified from p601-7 containing seven direct repeats of the 207 bp 601 nucleosome positioning sequence. To reconstitute nucleosome arrays, pG5ML601, which contains Gal4 binding sites (G5) linked to the adenovirus major late promoter (ML) proceeding 280 bp G-less cassette and, on both sites, seven direct repeats of the 601 nucleosome positioning sequence, was digested with EcoRI and HindIII, and the G5ML601 array DNA fragments were gel purified. Mononucleosomes and nucleosome arrays were reconstituted by mixing histone octamers and DNA templates at a ratio of 1:1.2 (w/w) and salt gradient dialysis, and purified by sedimentation in a 5-30% (vol/vol) glycerol gradient as described previously[1, 5] .

H3 Tail Peptide and Nucleosome Binding Assays

For in vitro binding assays, streptavidin-coated 96well plates were incubated with biotin-conjugated H3 tail peptides (200 ng) or biotin-labeled nucleosomes (200 ng) for 2 h at room temperature and washed with washing buffer (20 mM HEPES-KOH, pH 7.9, 0.5 mM EDTA, 150 mM KCl, 1 mM dithiothreitol, 10% glycerol, and 0.1% Nonidet P-40). Then the plates were incubated with Flag-tagged H1 subtypes (200 ng) in the presence of poly(dA-dT) (1 μg/ml) as a competitor for "nonspecific" DNA-protein interactions in binding buffer (20 mM HEPES-KOH, pH 7.9, 0.5 mM EDTA, 200 mM KCl, 1 mM dithiothreitol, 10% glycerol, and 0.1% Nonidet P-40) for 2 h at room temperature with gentle rotation. After washing of the plates, Flag antibody and secondary antibody incubations are followed by the addition of 50 μl TMB horseradish peroxidase substrate solution (Pierce) and development of the

colorimetric reaction. The reaction was stopped by addition of 50 µl sulfuric acid, and absorbance was read at 450 nm by a Plate Chameleon V plate reader. For fluorescence binding assays, FITC-conjugated H3 tail peptides (1 µg) and nucleosomes (1 µg) were mixed with GST-H1.2 (1 µg) immobilized on glutathione-Sepharose beads in buffers with increasing KCl concentrations. After 3 h incubation at 4 °C with gentle rotation, the supernatant was collected by a short low- speed centrifugation (e.g., 2000 g for 1 minute) to analyze H1.2 dissociation. The pellet was resuspended in 600 mM KCl buffer and centrifuged again to collect the bead-bound H1.2. The nucleosomes samples collected from the supernatant and beads were quantitated based on the fluorescence intensity measured by a Plate Chameleon V plate reader. To study the effects of H3K27me3 on H1.2-nucleosome interaction in vivo, control and EZH2-depleted MCF7 cells were permeabilized in low salt buffer (10 mM HEPES, pH 7.4, 10 mM KCl, 50 µg/ml digitonin and protease inhibitor cocktail), and nuclei were prepared essentially as described[6]. After washing with low salt buffer, the nuclei were treated with DNase I for 15 min at room temperature and an additional 15 min at 37 °C. Chromatin proteins were extracted with extraction buffer (1% Triton X-100, 50 mM HEPES, pH 7.4, 150 mM NaCl, 30 mM sodium phosphate, 10 mM NaF, 1 mM EDTA and protease inhibitor cocktail) for 10 min at 4 °C. Supernatant chromatin fractions were collected by centrifugation at 14,000 rpm for 10 min.

Restriction Enzyme Accessibility and Micrococcal Nuclease Digestion Assays
For restriction enzyme digestion assays, H3K27me0 and H3K27me3 601 nucleosomes (120 ng) were reconstituted on 5'-biotinylated 207 bp 601 nucleosome positioning sequence, incubated with Flag-H1.2 for 2 h at 4 °C, and immobilized via its 5' biotiny moiety on streptavidin-conjugated magnetic beads. Nucleosomes were digested for 2 h at 37 °C with 2 units of BsiEI and EagI, and the restriction digestions were terminated by adding stop buffer (20 mM Tris-HCl, pH 7.5, 50 mM EDTA, 1 M KCl, 2% SDS, and 1 mg/ml glycogen). Since the recognition sites of BsiEI and EagI are located around the 5' end of the nucleosomal DNA (see Figure 3A), digested nucleosomes are released from the beads. Supernatant containing free nucleosomes was treated with Proteinase K (0.2 mg/ml), extracted with phenol-chloroform, and precipitated with ethanol. The purified 601 DNA fragments were then analyzed by electrophoresis through 2.5% agarose gels. For MNase digestion assays, reconstituted G5ML601 nucleosome arrays (500 ng) were incubated with wild type or mutant H1.2 for 4 h, and treated with 0.001, 0.002, or 0.004 units of MNase (Sigma) at 37°C for 10 min. Subsequently, DNA from the MNase digestion was phenol-extracted, ethanol-precipitated, and analyzed on a 1 % agarose gel.

Gene Expression Microarray, qRT-PCR, and ChIP

Total RNA was isolated from two biological replicates of mock-, H1.2-, or EZH2-depleted MCF7 cells using the TRIzol reagent according to the manufacturer's instructions (Invitrogen). Gene expression microarray experiments were conducted using using a whole-genome expression array (Human HT-12 v4 Expression BeadChip, Illumina). Differential gene expression was detected by using the ArrayPipe software (www.pathogenomics.ca/arraypipe). Genes showing detection $p < 0.05$ with fold change > 2 were functionally analyzed in the context of gene ontology by using DAVID bioinformatics resources (http://david.abcc.ncifcrf.gov). Nonparametric GSEA was performed using GSEA (Broad Institute) as detailed previously47. For qRT-PCR, total RNA (2 µg) was reverse transcribed using the iScript cDNA Synthesis Kit (Bio-Rad) and the PerfeCta SYBR Green FastMix (Quanta biosciences) as recently described48. The primers used for qRT-PCR are summarized in Table S2. ChIP assays with MCF7 cells were performed using the ChIP assay kit (Millipore) as recently described48. Antibodies specific to H1.2, EZH2, H3K27me3, and H3 were used for immunoprecipitation. Immunoprecipitated DNA was analyzed with the primers that amplify the three different regions of the PGR, PTGES, XAF1 and OR8S1 loci. The primers used for qPCR are summarized in Table S3. The NCBI GEO accession number for microarray data reported in this paper is GSE68050.

SUPPLEMENTARY REFERENCES

1. Heo, K. *et al.* Isolation and characterization of proteins associated with histone H3 tails in vivo. *J. Biol. Chem.* **282**, 15476-15483 (2007).
2. Dyer, P.N. *et al.* Reconstitution of nucleosome core particles from recombinant histones and DNA. *Methods Enzymol.* **375**, 23-44 (2004).
3. Simon, M.D. *et al.* The site-specific installation of methyl-lysine analogs into recombinant histones. *Cell* **128**, 1003-1012 (2007).
4. Kim, K. *et al.* Isolation and characterization of a novel H1.2 complex that acts as a repressor of p53-mediated transcription. *J. Biol. Chem.* **283**, 9113-9126 (2008).
5. Kim, K. *et al.* Vpr-binding protein antagonizes p53-mediated transcription via direct interaction with H3 tail. *Mol. Cell. Biol.* **32**, 783-796 (2012).
6. Thomashevski, A. *et al.* The Fanconi anemia core complex forms four complexes of different sizes in different subcellular compartments. *J. Biol. Chem.* **279**, 26201-26209 (2004).

SUPPELEMNTARY FIGURE LEGENDS

Figure S1. H1.2 interaction with H3K27me3 tail peptide and nucleosome.
(A) Unmodified or mono-, di- or tri-methylated H3 tail peptides were fluorescent-conjugated and incubated with GST-fused wild type or mutant H1.2 immobilized on glutathione-Sepharose beads at the indicated KCl concentrations. The binding of H3 tail peptides to the H1.2 was measured with a fluorescence microplate reader. Data shown are representative of three independent experiments. (B) In vitro binding assays with Flag-H1 subtypes were performed essentially as described in Figure 1B, but immobilized H3K27me0 and H3K27me3 nucleosomes were used as indicated. (C) Nucleosome binding assays were conducted as described in Figure 1C, but using Flag-H1.2 proteins that were mutated at three H1.2-specific amino acids (V120, T126 and V132) in the C-terminal tail Of the input proteins, 10% were examined by Western blot. Sequence alignment of the putative H3K27me3-binding sequence in H1.2 (amino acids 110-145) with the corresponding sequences of other subtypes is shown in the upper panel. Identical and similar amino acids are highlighted in black and grey, respectively.

Figure S2. H1.4 mutant interaction with H3K27me3 nucleosome.
(A) H3K27me0 and H3K27me3 nucleosomes were reconstituted and immobilized on streptavidin beads as in Figure 1C. Flag-tagged wild-type or mutant H1.4 was incubated with immobilized nucleosomes, and their binding was analyzed by Western blot using Flag-antibody. Lanes 1 and 2 represent 10% of the input. Sequence alignment of the putative H3K27me3-binding motif in H1.2 and the corresponding regions of wild type and mutant H1.4 are shown in the upper panel. (B) Nucleosome binding assays were conducted as described in Figure 1E, but using GST-H1.4 wild type (wt) or GST-H1.4 A120V/A126T/A132V mutant (mt). Data shown are representative of three independent experiments.

Figure S3. Effects of EZH2/H1.2 knockdown on target gene expression and cell growth.
(A) Breast MCF7, bladder LD611 and prostate LNCaP cancer cell lines and their corresponding normal counterparts (MCF10-2A, LD419 and MLC) were lysed and subjected to Western blotting with the indicated antibodies. (B) H1.2-depleted MCF7, LD611 and LNCaP cancer cells were transfected with Flag-tagged form of wild type (wt) or V120/T126/V132-mutated (mt) RNAi-resistant H1.2, and whole cell extracts were analyzed by Western blotting with indicated antibodies. (C) EZH2-depleted MCF7, LD611 and LNCaP cancer cells were transfected with wild type (wt) or H689A-mutated (mt) RNAi-resistant EZH2, and subjected to Western blotting with indicated antibodies. (D and E) H1.2/EZH2-depleted LD611/LNCaP cancer cells were complemented with wild type or mutant H1.2/EZH2, and the mRNA levels of the three upregulated genes were quantified by qRT-PCR using primers listed in Supplementary Table S2. Data represent the mean ± SD of three independent experiments. (F and G) H1.2/EZH2-depleted LD611/LNCaP cancer cells were complemented with wild type or mutant H1.2/EZH2, and cell proliferation was measured over a period of four days by MTT assays as indicated. Data represent the mean ± SD of three replicates in three independent experiments.

Figure S4. Changes in H1.2 and H3K27me3 levels at target loci after H1.2 and EZH2 knockdown.
(A) Diagram of target genes showing ChIP amplicon locations. (B-G) H1.2/EZH2-depleted MCF7 cells were complemented with wild type or mutant H1.2/EZH2, and ChIP assays of two target genes (*PTGES* and *XAF1*) and one control gene (*OR8S1*) were performed using H3K27me3, H1.2, EZH2 and H3 antibodies. The precipitated DNA was quantified by qPCR with the primers listed in Table S3. Data represent the mean ± SD of three independent experiments.

Figure S5. Effects of H1.4 on target gene expression and H1.2/H3K27me3 enrichment.
(A) H1.2/EZH2-depleted MCF7 cells were transfected with H1.4, and analyzed by Western blotting with indicated antibodies. (B) H1.2-depleted MCF7 cells were transfected with Flag-H1.2 or Flag-H1.4 and analyzed by Western blotting with H1.2 and actin antibodies. (C) H1.2/EZH2-depleted MCF7 cancer cells were transfected with H1.4, and the mRNA levels of three target genes (*PGR*, *PTGES* and *XAF1*)

were quantified by qRT-PCR. (D) H1.2/EZH2-depleted MCF7 cells were transfected with H1.4, and ChIP assays of the three target genes were performed using H3K27me3, H1.2 and Flag antibodies.

Figure S6. Analysis of EZH2-dependent chromatin reorganization by H1.2.
(A) GFP-fused wild type and mutant versions of H1.2 were expressed in MCF7 cells, and representative confocal images of GFP-H1.2 and nuclear DAPI staining of the cells are shown on the left panel. The right panel graphically shows the increase of heterochromatin in cells transfected with wild type and mutant H1.2. The results shown are representative of three independent experiments. (B) DNA FISH analyses were identical to Figure 6B, except that two probes (~170 kb in length) recognizing two control loci in the chromosome 12 were used. The majority of the two loci were spatially close in mock-depleted MCF7 cells, and knockdown/re-expression of EZH2/H1.2 exhibited little to no change in the spatially close localization of the two loci. (C-E) Nuclease protection assays were performed as in Figure 6C, but using overlapping amplicons specific for the promoter regions (-1.5 kb-TSS) of two target genes (*PTGES*, *XAF1*) and one control gene (*OR8S1*). The extent of MNase digestion was determined based on the enrichment of digested DNA relative to sonicated genomic DNA at each location. Data represent the mean ± SD of three independent experiments.

Table S2. Primer sequence for qRT-PCR.

Primers	Forward (5'-3')	Reverse (5'-3')
ACTB	TCACCGAGCGCGGCT	TAATGTCACGCACGATTTCCC
PGR	TGAAAGGACAGTTTCACCTTCTCG	CACCTTGCTCCTCATTTCTGAGTG
PTGES	GAAGAAGGCCTTTGCCAAC	GGGTTAGGACCCAGAAAGGA
XAF1	TGCAACCAAATGATTCCAGA	TTTCAGCAGCTTGACTTGGA
EI24	CGTCGAAGAAGGGCAAGTAG	CACCATTCCAAGCACAACAC
SOCS2	AAAAGAGGCACCAGAAGGAA	GTCCGCTTATCCTTGCACAT
APIP	ACTGGGACTGGAGGAGGAAT	ATCACTGCACCTGCTCCTCT
EIF2AK2	ACAATTGGCCGCTAAACTTG	GCGAGTGTGCTGGTCACTAA
IFI6	AAGGCGGTATCGCTTTTCTT	TTCTTACCCGCATTCTCACC
OR8S1	CCTGGCTCAGGTCTTCTTTG	TGATGAGTGCGTCCAGAAAG
H1.2	ACGAGCTTTGCCACTTGTACC	TGGCCGCCTTCTTCTTTACAGG
H1.4	GTCGGGTTCCTTCAAACTCA	GCCTTCTTTGGGGTCTTCTT
GAPDH	GGCCTCCAAGGAGTAAGACC	AGGGGAGATTCAGTGTGGTG

Table S3. Primer sequence for qPCR.

Primers	Forward (5'-3')	Reverse (5'-3')
PGR-PR	CCAATGGTCTTGGGTCAAAT	AATGGATCCCCGCTAAATTC
PGR-TSS	TCGGGGTAAGCCTTGTTGTA	GCCTCGGGTTGTAGATTTCA
PGR-CR	GACTGAGCTGAAGGCAAAGG	CGAAACTTCAGGCAAGGTGT
PTGES-PR	AGGTAATCTGTCCCCCTTGG	GGCTCCACTGACTTCCTGAG
PTGES-TSS	CGGCAACTGCTTGTCTTTCT	TGTGATCAGCTCGACAGAGG
PTGES-CR	TCTTCCCCTGTGTCCAGTTC	AGATCCTCACTCCCCAGGAT
XAF1-PR	TCTCGAACTCCTGACCTCGT	TGACCCTCACTACCCACTCC
XAF1-TSS	CAGCCTCAGGGAGGTAGATG	CTCCCAAGAAGCCCCTTTAC
XAF1-CR	TCAGGCAGAGGAGAGAGAGC	TCCATCAGCTCTTGCTTGTG
OR8S1-PR	CAGGAGTGGGCTTCTACCAG	TTTCTGCTAATGCTGCTGTTG
OR8S1-TSS	ATCACCGAGTTCCTCCTCCT	TAAATCCCCAGGAACAGCAC
OR8S1-CR	GGTGAAACCCTGTCTCCAAA	CAAGTGATCCTCCTCCCTCA

Table S4. Primer sequence for MNase-qPCR.

Primers	Forward (5'-3')	Reverse (5'-3')
PGR-R1	GTACGGAGCCAGCAGAAGTC	TCTCAGTCCCTCGCTGAGTT
PGR-R2	AACTCAGCGAGGGACTGAGA	GAGGACTGGAGACGCAGAGT
PGR-R3	GGAGAACTCCCCGAGTTAGG	AGGGAGGAGAAAGTGGGTGT
PGR-R4	TCCCTCTGCCCCTATATTCC	CGAAACTTCAGGCAAGGTGT
PGR-R5	ACACCTTGCCTGAAGTTTCG	TCCAAGACACTGTCCAGCAG
PGR-R6	TGCTGGACAGTGTCTTGGAC	AGCTGTCTCCAACCTTGCAC
PGR-R7	CTCTGAGAGCCCTCACTGGT	GAGAAGCGGGAATCTTCCTT
PTGES-R1	GTGTCTGCCATCACATCTGG	CCCTCACCTCAAAATCCTCA
PTGES-R2	GCCAAGGTGAGGATTTTGAG	CCATGTTCTCACCACTGCAC
PTGES-R3	GTGCAGTGGTGAGAACATGG	CCAAGGGGGACAGATTACCT
PTGES-R4	AGGTAATCTGTCCCCCTTGG	GGCTCCACTGACTTCCTGAG
PTGES-R5	TGAAGCCTGAAATGGGTCTC	GGAGAGACGCTAACCTGCTG
PTGES-R6	TCCAAGAGGGAGGCAGTAAA	CTTCAAGGCCAGCAGGTTAG
PTGES-R7	TTTACTGCCTCCCTCTTGGA	GGCAAAAGGGACTTTTCACA
XAF1-R1	CCTATCCCATCTCAGCAGGA	AGCTACTCGGGAGGCTAAGG
XAF1-R2	GCCAGGATGGTCTCAATCTC	ATTGCTTGAACCCAGGAGGT
XAF1-R3	GTAGAGACGGGGTTTCACCA	TGACCCTCACTACCCACTCC
XAF1-R4	GGAGTGGGTAGTGAGGGTCA	CTAGGCCAATCGCTCAACAT
XAF1-R5	CTAGCCTCTCTCTGCCTCCA	TTCCAGGCACCATTCTAAGC
XAF1-R6	AATGGTGCCTGGAACATAGG	CGCCTCTCTGAGGAGGATAA
XAF1-R7	TTATCCTCCTCAGAGAGGCG	CTCTGGGGGTCCTTAGAGTT
OR8S1-R1	AGGCATTCCTGGGATACACA	GTGGGAGAGAAGTTGGTTGG
OR8S1-R2	TGCCCTCCTTTCTGAATGAC	CTGGTAGAAGCCCACTCCTG
OR8S1-R3	CAGGAGTGGGCTTCTACCAG	TTTCTGCTAATGCTGCTGTTG
OR8S1-R4	AGGTAGTTTGAAGCGCCTTAT	GCCACACAGATCAATGGCTA
OR8S1-R5	CCATTGATCTGTGTGGCTCA	TGGCATGAAGAATGTGTTCG
OR8S1-R6	TTTCCTCAGTCTTGAGTGTTCG	ACTCCAGTCTGGGCAACAAG
OR8S1-R7	AGTGATTCTCCTGCCTCAGC	GAGTTCGAGACCAGCCTGAC

Supplementary Figure S1

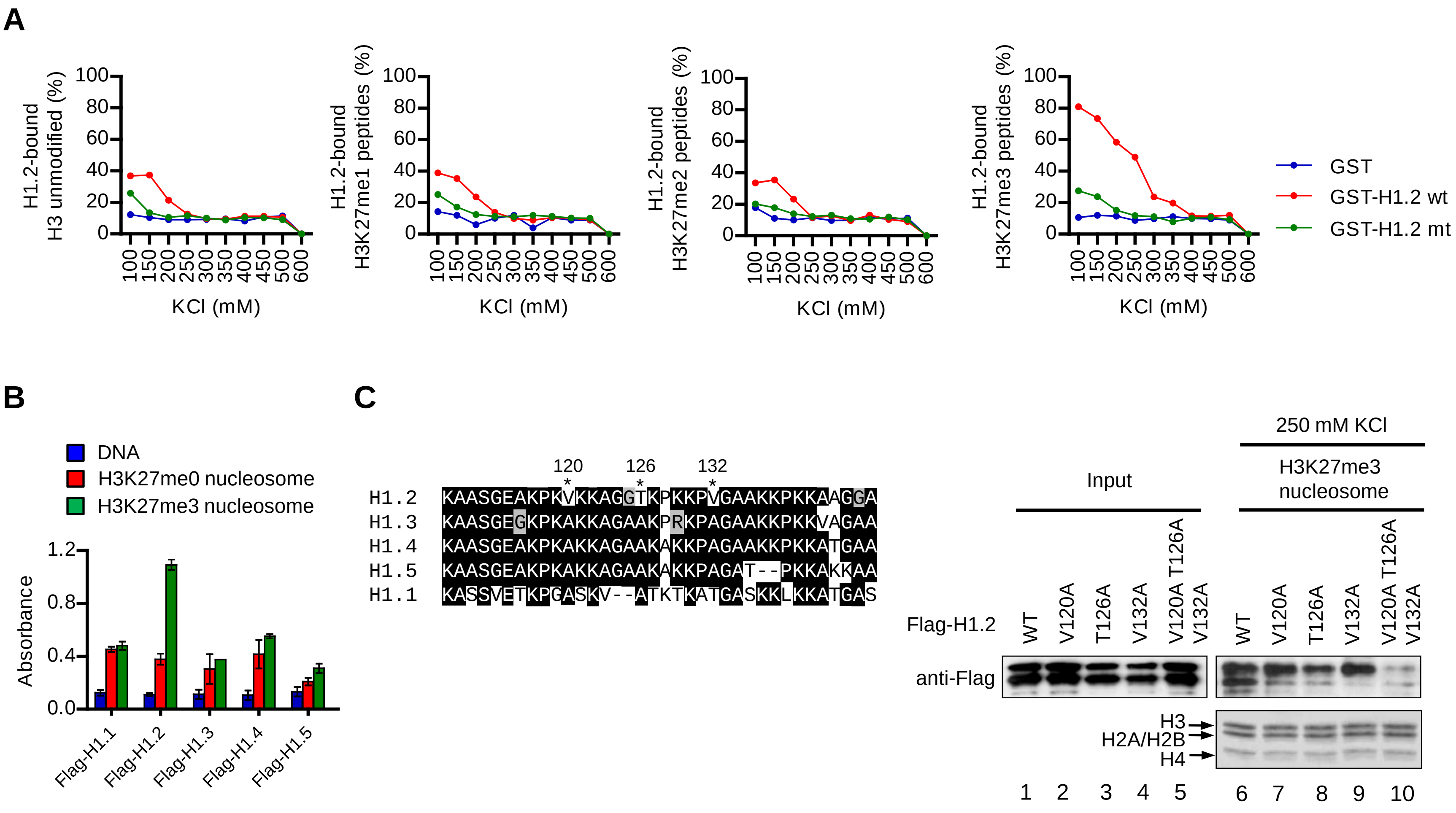

Supplementary Figure S2

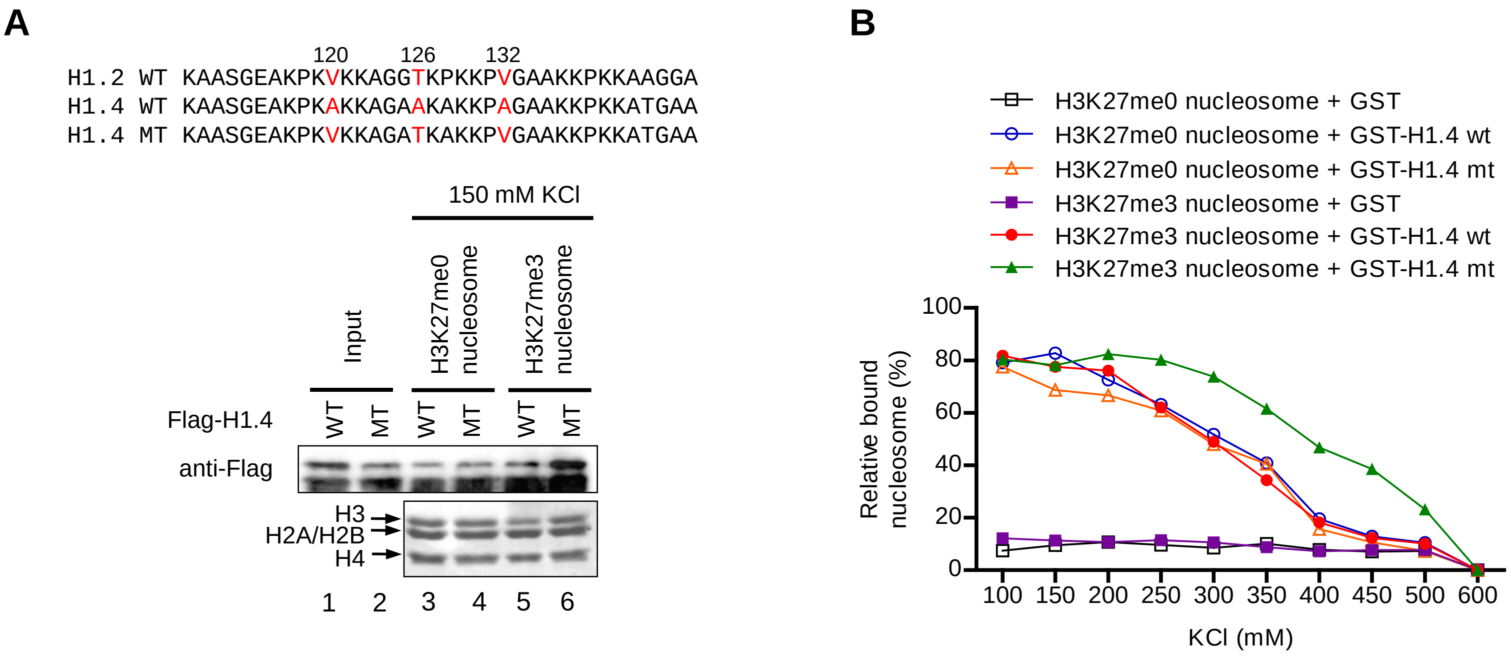

Supplementary Figure S3

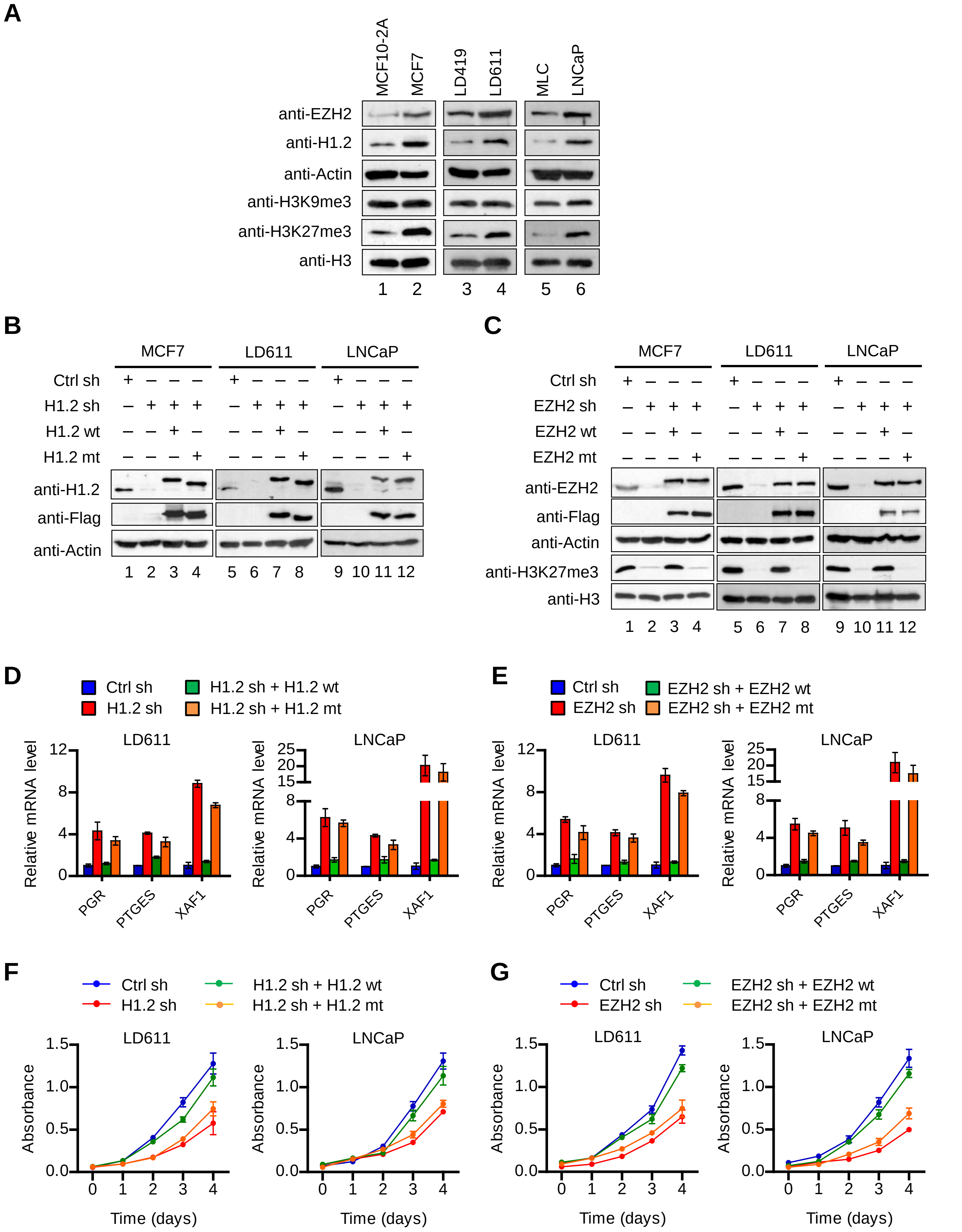

Supplementary Figure S4

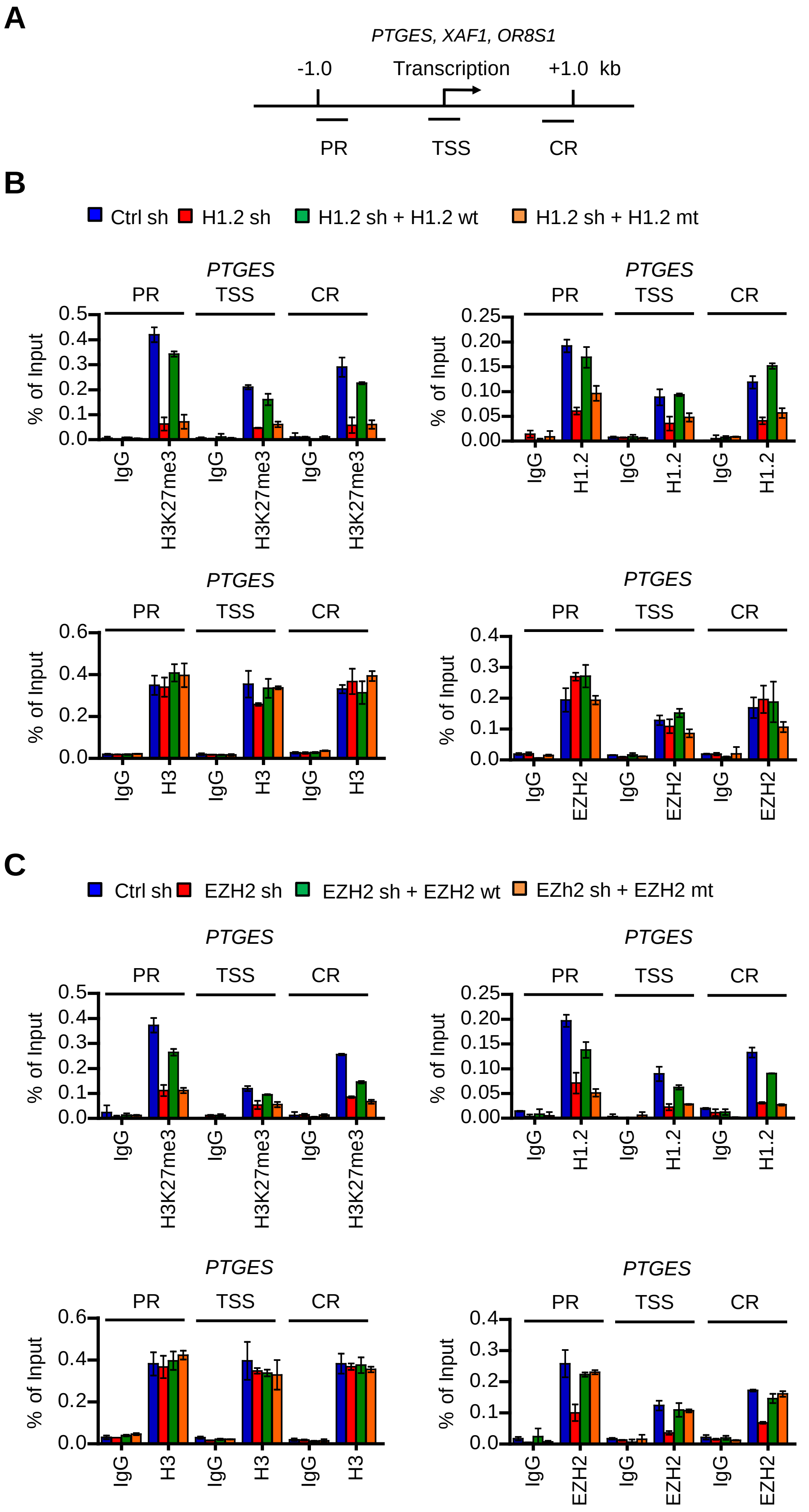

Supplementary Figure S4

D

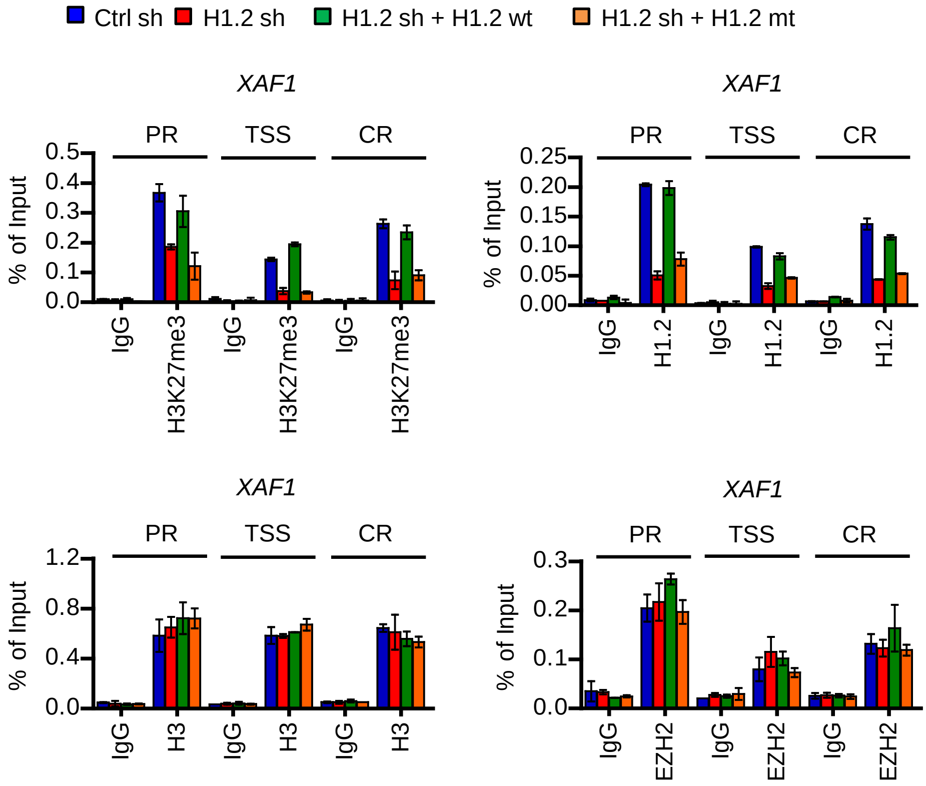

E

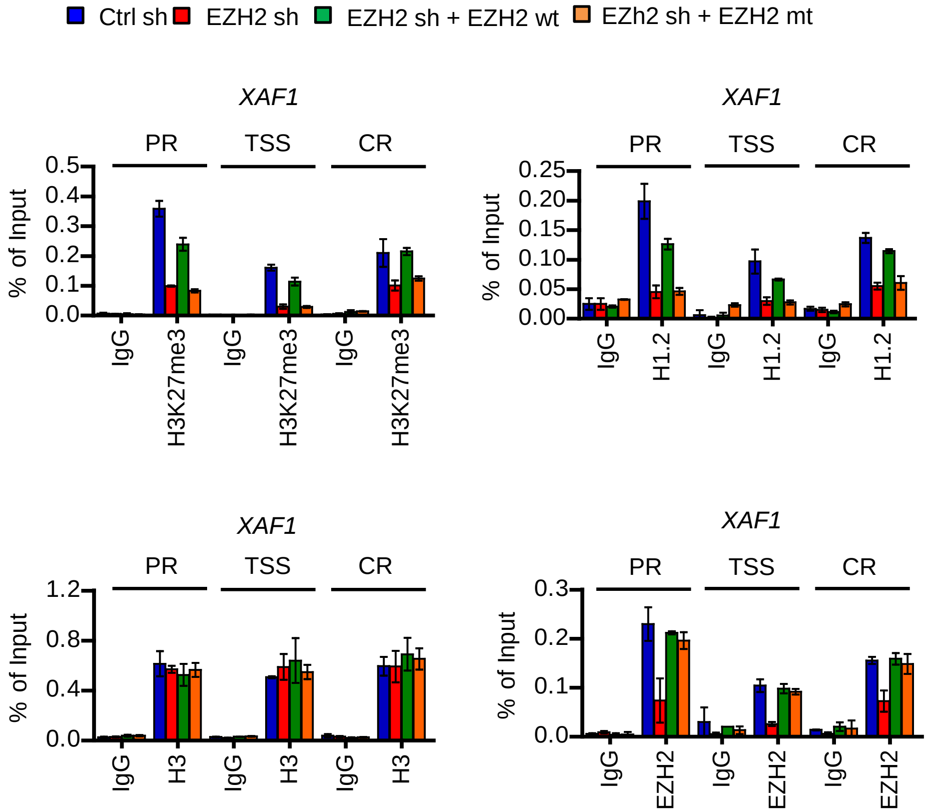

Supplementary Figure S4

F

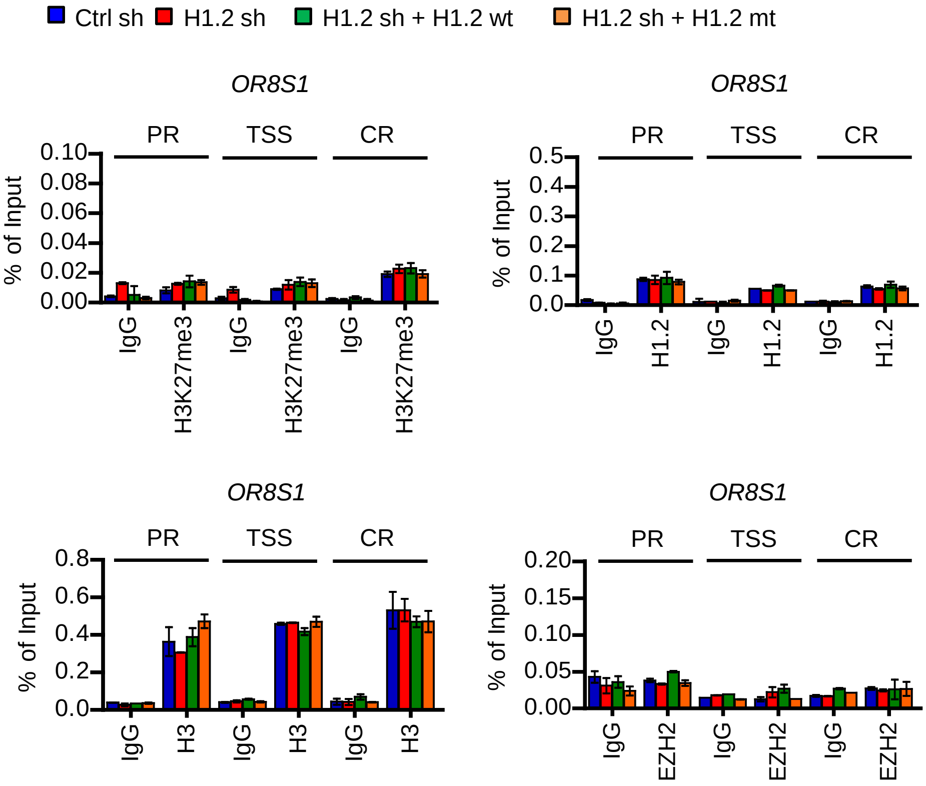

G

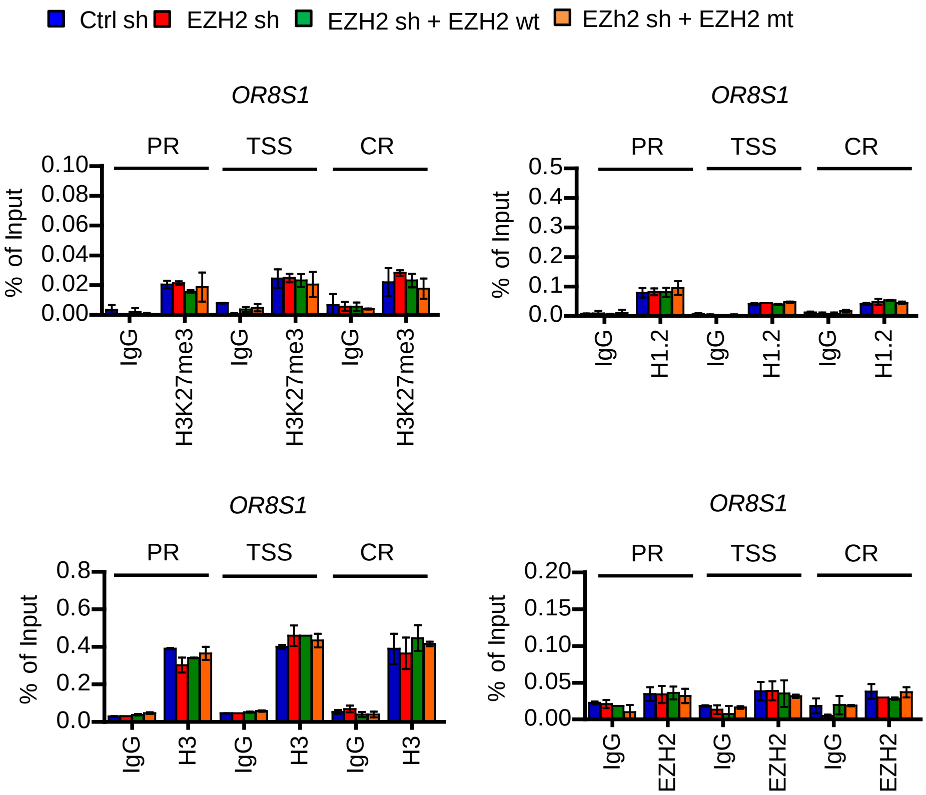

Supplementary Figure S5

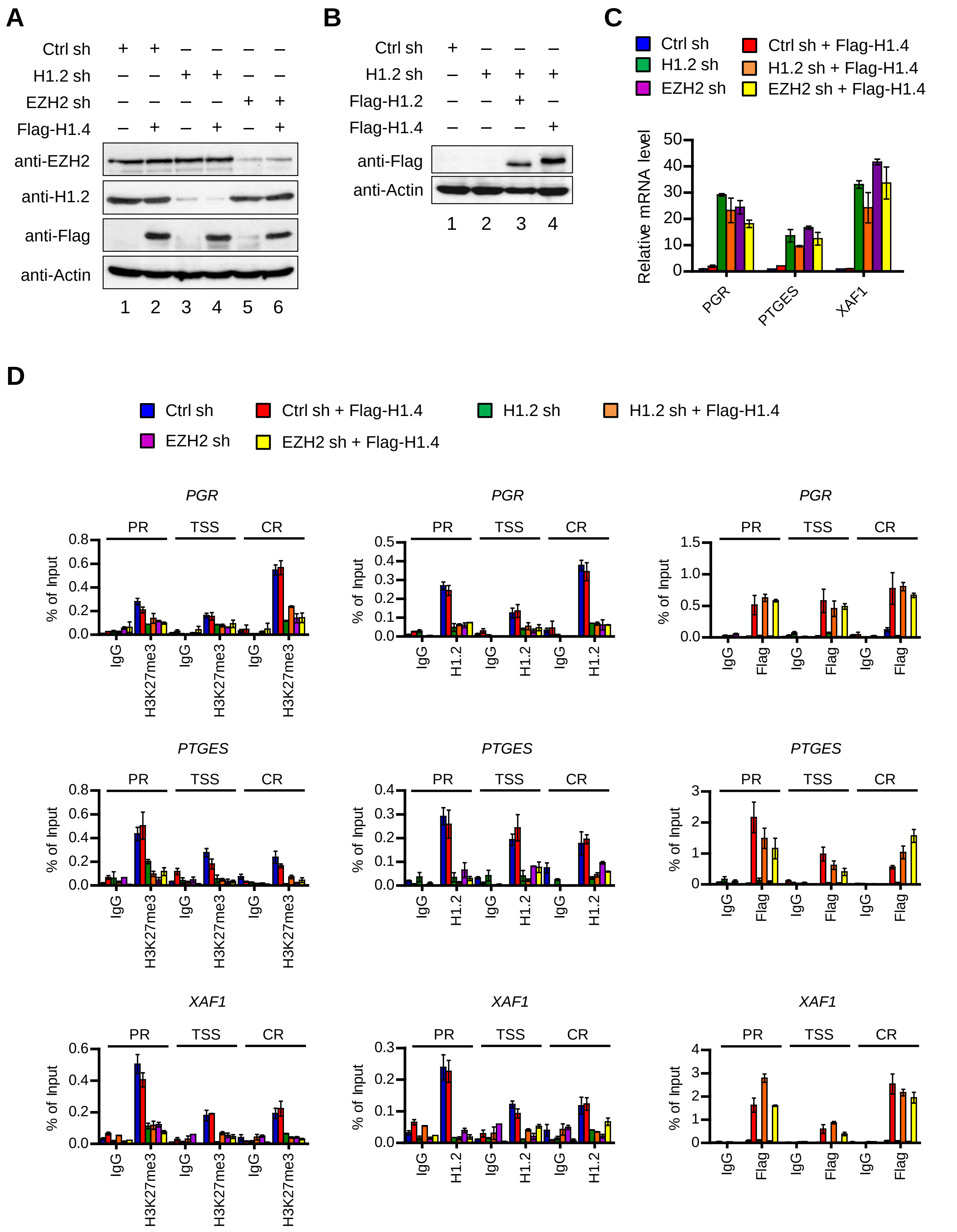

Supplementary Figure S6

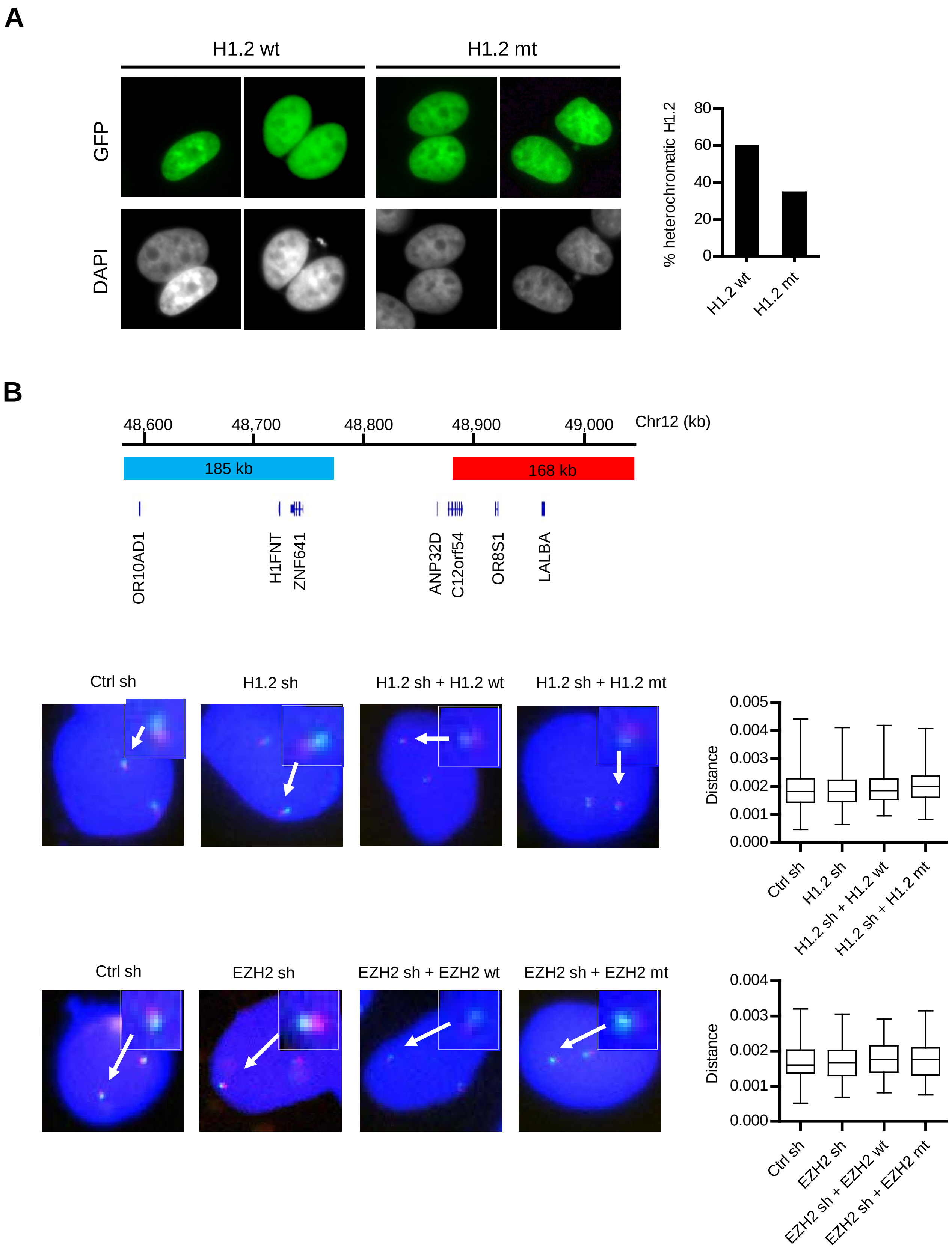

Supplementary Figure S6

C

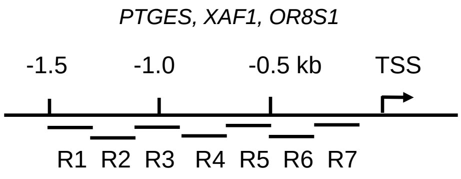

D

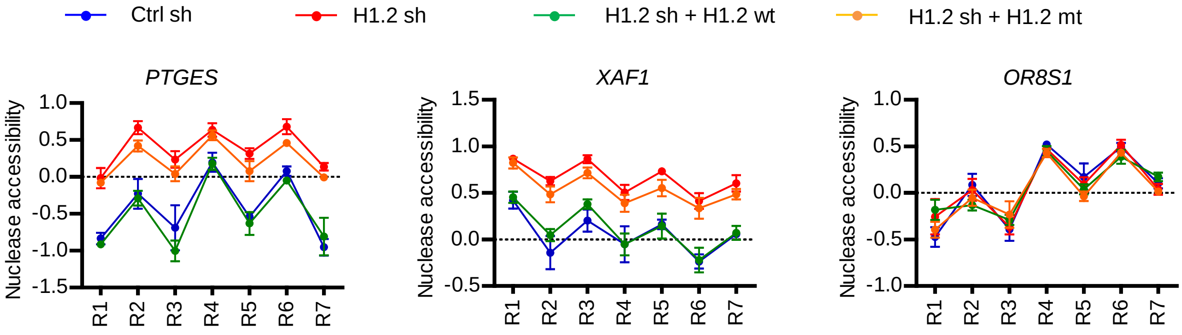

E

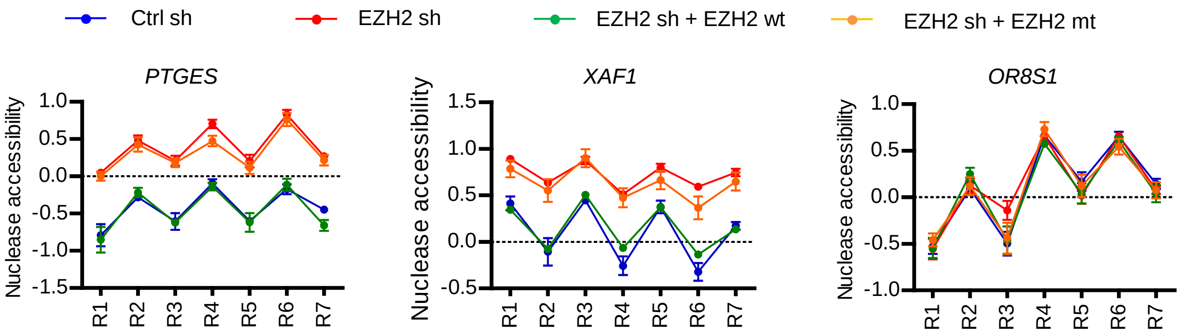

Supplement: Supplementary Information [file srep16714-s1.pdf]
